# Strengthening monocarboxylate transporters by adiponectin receptor agonist ameliorates diabetic peripheral neuropathy

**DOI:** 10.1186/s12964-025-02326-5

**Published:** 2025-07-16

**Authors:** Tae Woo Kim, Sae-Jong Oum, Ji Hee Lim, Yaeni Kim, Eun Nim Kim, Yongjie Jin, Yu Ah Hong, Seung Yun Chae, Bum Soon Choi, Hye Won Kim, Cheol Whee Park

**Affiliations:** 1 Division of Nephrology, Department of Internal Medicine, College of Medicine, The Catholic University of Korea, Seoul, Republic of Korea; 2 Institute for Aging and Metabolic Diseases, College of Medicine, The Catholic University of Korea, Seoul, Republic of Korea; 3 Department of Rehabilitation Medicine, Bucheon St. Mary’s Hospital, College of Medicine, The Catholic University of Korea, Bucheon, Republic of Korea; 4 Division of Nephrology, Department of Medicine, The Fifth Affiliated Hospital of Sun Yat-Sen University, Zhuhai, 519000 Guangdong China; 5 Division of Nephrology, Department of Internal Medicine Seoul St. Mary’s Hospital, College of Medicine, The Catholic University of Korea, 222, Banpo-daero, Seocho-gu, Seoul, 06591 Republic of Korea

**Keywords:** Adiponectin, Diabetic peripheral neuropathy, Glucolipotoxicity, Monocarboxylate transporter

## Abstract

**Background:**

Disruption of energy support to peripheral nerves leads to dysfunction and degeneration of both neuronal axons and Schwann cells (SCs).

**Methods:**

We evaluated the effects of the adiponectin receptor (AdipoR) agonist, AdipoRon on diabetic peripheral neuropathy (DPN) using *db/db* mice, murine ND7/23 cells, and human SCs.

**Results:**

AdipoRon improved sensorimotor function and restored nerve phenotypes in the sciatic nerve and paw skin of *db/db* mice by reducing systemic oxidative stress and insulin resistance. AdipoRon restored impaired oxidative stress response, apoptosis, and autophagy activity through increased AdipoR1/R2-intracellular Ca^++^-CaMKKβ expression as well as LKB1/AMPK-PPARα/PGC-1α/Nrf2 phosphorylation. It also decreased mTOR phosphorylation, which is related to the preservation of mitochondria in axons and SCs, and improved MCT1/2/4 expression and lactate and ATP/AMP levels in the sciatic nerve of *db/db* mice. In ND7/23 cells and SCs, AdipoRon decreased oxidative stress and apoptosis while increasing autophagy by suppressing high glucose- and palmitate-induced cellular and mitochondrial oxidative stress, activating the same signaling as in the sciatic nerve. These protective changes were induced by providing energy substrate, lactate and ATP, through the increased expression of MCT1/2/4, facilitating metabolic communication between neurons and SCs.

**Conclusion:**

AdipoRon may play an important role in preventing DPN by ameliorating oxidative stress, apoptosis, and autophagy, strengthening metabolic support for neurons and SCs under diabetic conditions.

## Introduction

Diabetic peripheral neuropathy (DPN), characterized by length-dependent damage to peripheral nerves, is one of the most common microvascular complications of diabetes [1, 2]. It is clinically important due to its association with high mortality and poor prognosis. Approximately 30% of diabetes patients have DPN at diagnosis, and 50% develop it over the disease course [3]. The key risk factors for DPN include hyperglycemia [4], hyperlipidemia [5], and hypertension [6]. These factors contribute to DPN through oxidative stress, inflammation, endoplasmic reticulum stress, and mitochondrial dysfunction, ultimately leading to irreversible nerve damage, neurodegeneration, and apoptotic cell death [7]. The only proven treatment for delaying the onset or progression of DPN is intensive glycemic control. However, DPN often develops and progresses in most patients despite this approach [8].

The precise mechanism and sequence of cellular injury in DPN are not fully understood but likely involve abnormalities in both vascular function and cellular metabolism of peripheral nerves. It is unclear whether damage to neuronal axons or axon-supported Schwann cells (SCs) occurs before damage to neuronal cell bodies [3]. These changes lead to altered axon-SC transport, demyelination, degeneration, apoptosis, autophagy, and the disruption of cellular metabolism in neurons and SCs. DPN represents a state of impaired metabolism and mitochondrial bio-energetic failure within long peripheral nerve axons supported by SCs [1]. Axon-SC interactions are crucial for maintaining normal sensorimotor function and for transporting molecules and organelles along the axon. Disruption of axon-SC metabolic interactions in diseased peripheral nerves may be both a cause and a consequence of pathology [9].

Monocarboxylate transporters (MCTs) are responsible for transporting nutrients such as lactate, pyruvate, acetoacetate, and β-hydroxybutyrate, as well as for cellular mechanism and pH regulation in peripheral nerves [10]. MCT1, MCT2 and MCT4 are expressed in SCs, with MCT1 present in the myelin sheath and Schmidt-Lanterman incisures and MCT1 and MCT2 detected in the dorsal root ganglion (DRG) [11]. MCT1 is the primary transporter for lactate in the peripheral nervous system (PNS) and is critical for peripheral nerve regeneration and metabolic support to neuronal axons [12] and SCs [13]. Despite a large interest in MCT2 in SCs and nerve cells, its role in disease remains poorly understood. A recent study showed that MCT2 overexpression reduces metabolic vulnerability and protects retinal ganglion cells, suggesting that enhanced MCT2 overexpression improves energy homeostasis and may help combat neurodegeneration [14].

Recent research has highlighted the critical role of the crosstalk between PNS and adipocytes in peripheral nerve regeneration following acute nerve trauma [15]. Adipocyte-derived leptin supports peripheral nerve repair through leptin receptor signaling in SCs and modulates catabolic processes including mitochondrial respiration and autophagy. Adiponectin, another important adipokine secreted primarily by adipocytes, regulates energy homeostasis as well as glucose and fatty acid metabolism through adiponectin receptors (AdipoR)1 and 2 [16]. Studies suggest that reduced adiponectin levels may contribute to diabetic foot ulcers, whereas adiponectin promotes wound healing in diabetes through its antioxidative, anti-inflammatory, anti-fibrotic, and anti-apoptotic effects [17]. Pharmacological activation of AdipoRs by the synthetic agonist AdipoRon has been shown to promote wound healing [18] and provide renal and cardioprotective benefits through AMP-activated protein kinase (AMPK) and peroxisome proliferator-activated receptor (PPAR)α pathways, which regulate energy homeostasis and fatty acid β-oxidation [19, 20]. This suggests a potential role for AdipoR agonists in preventing DPN by modulating cellular metabolism in neurons and SCs. However, previous studies investigating the effects of serum adiponectin on DPN have yielded inconsistent results. Some studies reported higher serum adiponectin levels in patients with DPN than that in non-DPN patients [21], while others found that low serum adiponectin levels were significantly associated with DPN incidence [22]. Furthermore, some studies have suggested that there is no relationship between adiponectin and DPN [23]. Adiponectin has been shown to increase mitochondrial biogenesis and oxidative capacity in skeletal muscle [24, 25] through AMPK activation and peroxisome proliferator–activated receptor γ coactivator 1α (PGC-1α) [24–26]. Under diabetic conditions, decreased fatty acid oxidation leads to adenosine triphosphate (ATP) depletion, accompanied by changes in mitochondrial number and morphology [27]. Mitochondrial function in SCs is essential for maintaining axonal survival and normal peripheral nerve function [28].

In this study, we examined AdipoR1/R2 expression in the sciatic nerves of in type 2 diabetic *db/db* mice, which are homozygous for diabetes spontaneous mutation *Lept*^*db*^, to eliminate the effects of leptin following AdipoRon treatment. We hypothesized that AdipoRon could ameliorate DPN in the sciatic nerve and paw skin of *db/db* mice by maintaining normal functional and morphological patterns. This improvement may be achieved by increasing the expression of AdipoR1/R2, activating their target molecules through the AMPK/PPARα pathway, and suppressing the mammalian target of rapamycin (mTOR). Additionally, associated downstream pathways including PGC-1α/the nuclear factor erythroid 2- related factor 2 (Nrf2) could be involved with the support of MCT1/2/4 in nerve cells and SCs. These changes could lead to a decrease in oxidative stress, especially in mitochondria, which is main source of reactive oxygen species (ROS), and an increase in mitochondrial efficiency, enhancing energy interactions between axons and SCs primarily through lactate shuttling and ATP production [29].

## Methods and materials

### Experimental animals and assessment of peripheral nerve function

Eight-week-old male C57BLKS/J *db/m* and *db/db* mice were obtained from Jackson Laboratories (Bar Harbor, ME, USA). The *db/m* and *db/db* mice were divided into four groups and were provided either with a standard diet chow or a diet containing AdipoRon. AdipoRon (30 mg/kg; Sigma, St Louis, MO) was mixed into the standard chow diet and provided to *db/db* mice (*db/db* + AdipoR, *n* = 8) and age- and gender-matched *db/m* mice (*db/m* + AdipoR, *n* = 8) from 16 weeks of age for 4 weeks. Control *db/db* (*db/db* cont, *n* = 8) and *db/m* mice (*db/m* cont, *n* = 8) were provided with standard diet chow. Accordingly, only male mice were used in this study, based on previous reports showing that male *db/db* mice exhibit more severe diabetic neurovascular and cognitive impairments compared to females [30]. Following 4 weeks of AdipoRon treatment, we performed electrophysiological and sensory threshold tests in the following sequence: tactile responses (50% response rate when the tip is applied to the hind paw) using flexible von Frey filaments, followed by sciatic motor nerve conduction latency (MNCL), as described previously [31]. Finally, the mice were euthanized with 10 mg/kg xylazine hydrochloride (Rompun; Bayer, Leuverkusen, Germany) and 30 mg/kg tiletamine plus zolazepam (Zoletil; Virbac, Carros, France). Blood samples were collected from the left ventricle, centrifuged at 2000 x g for 10 min to obtain blood serum, and the serum was stored at − 70 °C for subsequent analyses. Sciatic nerve and hind limb footpad skin were also dissected for further analyses. All animal procedures were carried out in compliance with the Laboratory Animals Welfare Act, the *Guide for the Care and Use of Laboratory Animals*, and were approved by the Institutional Animal Care and Use Committee (IACUC) at College of Medicine, the Catholic University of Korea (CUMC-2017 − 0251-01).

### Assessment of biochemical and physical characteristics

During the experimental period, body weight was evaluated once a week. After 4 weeks of AdipoRon treatment, blood glucose was measured using an Accucheck meter (Roche Diagnostics, St. Louis, MO). Hemoglobin A1c (HbA1c) was measured in red cell lysates using an autoanalyzer (Bayer Healthcare, LLC, IN). Plasma insulin concentrations were measured by RIA (Alpco, Salem, NH), and the HOMA-IR index was calculated as follows: fasting glucose (mmol/L) × fasting insulin (mU/L)/22.5 [32]. Total cholesterol (FUJIFILM Wako Pure Chemical Corporation, 294-65801, Tokyo, Japan), triglyceride (FUJIFILM Wako Pure Chemical Corporation, 290-63701), and nonesterified fatty acid (FUJIFILM Wako Pure Chemical Corporation, 294-63601) concentrations were measured using commercial assay kits. The concentration of serum adiponectin was determined by ELISA (Biosource, Camarillo, CA). To assess oxidative stress, we measured the 24-hour urinary 8-hydroxy-deoxyguanosin (8-OHdG; OXIS Health Products, Inc. Portland, OR), urinary isoprostane (OXIS Health Products, Inc., Portland, OR). Furthermore, lactate (Cell Biolabs, Inc. of San Diego, CA), AMP (Abcam, Cambridge, UK), and ATP (Abcam, Cambridge, UK) levels were measured in serum samples using commercial assay kits according to the manufacturer’s instructions.

### Histological analysis

Sciatic nerve and hind limb footpad skin tissues were fixed in 4% paraformaldehyde, embedded in paraffin, and sectioned at 4 μm thickness. Immunofluorescence (IF) staining was performed to evaluate oxidative stress, extracellular matrix changes, metabolic enzyme expression, and neural injury in a tissue-specific manner. In sciatic nerve tissues, 8-hydroxy-deoxyguanosine (8-OHdG; JalCA, Japan) and type IV collagen (Col IV; Biodesign International, USA) were individually stained to assess oxidative DNA damage and extracellular matrix alterations, respectively, along with total Nrf2 (Proteintech, USA). In footpad skin, double IF staining for 8-OHdG and Col IV was performed to evaluate oxidative stress in the dermal matrix. To examine metabolic alterations in the sciatic nerve, IF staining was conducted for LDHA (Proteintech, USA) and LDHB (Proteintech, USA). For analysis of lactate transporter localization, triple IF staining was performed using antibodies against MCT1, MCT2, or MCT4 (Proteintech, USA), neurofilament light chain (NF-L; Santa Cruz Biotechnology, USA) as an axonal marker, and myelin basic protein (MBP; MyBioSource, USA) as a myelin marker. Apoptosis and autophagy were assessed by TUNEL staining (ApopTag Fluorescein In Situ Apoptosis Detection Kit; Millipore, USA) and LC3 antibody (Sigma-Aldrich, USA), respectively, in both sciatic nerve and footpad skin. To confirm neuronal specificity, double IF staining was performed with β3-tubulin (Cell Signaling Technology, USA), a neuronal marker, together with TUNEL or LC3. In footpad skin, Masson’s trichrome staining (Leica Microsystems) was used to evaluate tissue fibrosis, and the collagen-positive (blue-stained) area was semi-quantitatively analyzed using ImageJ software. Subsequently, Schwann cells and intraepidermal nerve fibers were identified by IF staining with SOX10 (Abcam, Cambridge, UK) and PGP9.5 (Abcam, Cambridge, UK) antibodies, respectively. All fluorescent images were acquired using a laser scanning confocal microscope (Carl Zeiss LSM 900, Germany), and histological images were examined under bright-field microscopy (Olympus BX50, Japan).

### Electron microscopy

For transmission electron microscopy (TEM), sciatic nerve specimens were fixed in 4% paraformaldehyde and 2.5% glutaraldehyde in 0.1 M phosphate buffer overnight at 4 °C. After washing in 0.1 M phosphate buffer, the specimens were post-fixed with 1% osmium tetroxide in the same buffer for 1 h. The specimens were then dehydrated in a graded series of ethanol, followed by acetone, and embedded in Epon 812. Ultrathin Sects. (70–80 nm) were cut using an ultramicrotome (Leica Ultracut UCT, Leica, Germany) and double-stained with uranyl acetate and lead citrate. These sections were examined with a transmission electron microscope (JEM 1010, Tokyo, Japan) at 60 kV. We measured areas of unmyelinated fibers, axonal diameters, *G* ratios, the number of degenerative fibers, the number of mitochondria in myelinated cells, and the number of mitochondria in Schwann cells using NIH Image J.

### Western blot analysis

Total proteins were extracted from sciatic nerve tissues, ND7/23 cell lines, and Human Schwann cells (HSCs). Tissues were homogenized, and cells were lysed in Pro-Prep Protein Extraction Solution (Intron Biotechnology, Gyeonggi-Do, Korea) supplemented with protease and phosphatase inhibitors (Sigma-Aldrich, St. Louis, MO). For ND7/23 cell lines and HSCs, cytoplasmic and nuclear proteins were additionally separated using the NE-PER Nuclear and Cytoplasmic Extraction Kit (Thermo Fisher Scientific, Waltham, MA). Protein concentrations were determined using the BCA Protein Assay Kit (Thermo Fisher Scientific, Waltham, MA). Equal amounts of protein (10 µg) were separated by SDS-PAGE on 10% polyacrylamide gels and transferred to nitrocellulose membranes (Millipore, Billerica, MA). Membranes were blocked with 3% non-fat dry milk in TBST for 1 h at room temperature and then incubated overnight at 4 °C with primary antibodies. The primary antibodies targeted AdipoR1, AdipoR2, CaMMKα (Santa Cruz Biotechnology, Santa Cruz, CA), CaMKKβ (Proteintech, Rosemont, IL), total LKB1, phospho-Ser428 LKB1 (pLKB1), total AMPK, phospho-Thr172 AMPK (pAMPK), total mTOR, phospho-Ser2448 mTOR (p-mTOR; Cell Signaling Technology, Danvers, MA), PPARα (Abcam, Cambridge, UK), total Nrf2 and phospho-Ser40 Nrf2 (GeneTex, Irvine, CA), PGC-1α (Novus Biologicals, Littleton, CO, USA), LC-3 (Sigma-Aldrich, St. Louis, MO), LDHA and LDHB (Proteintech, Rosemont, IL), MCT1, MCT2, MCT4 (Proteintech, Rosemont, IL), and GAPDH (Santa Cruz Biotechnology, Santa Cruz, CA) across all samples. Additionally, Bcl-2 and Bax (Santa Cruz Biotechnology, Santa Cruz, CA) were analyzed in sciatic nerve tissues and ND7/23 cell lines, while cytoplasmic and nuclear Nrf2 (GeneTex, Irvine, CA), along with nuclear lamin B (GeneTex, Irvine, CA), were analyzed in ND7/23 cell lines and HSCs. After washing with TBST, the membranes were incubated with HRP-conjugated secondary antibodies (Cell Signaling Technology, Danvers, MA) for 2 h at room temperature. Protein bands were visualized using an enhanced chemiluminescence (ECL) detection system (GE Healthcare, Chicago, IL) and quantified using ImageJ software (NIH, Bethesda, MD). The relative expression levels of the target proteins were normalized to the levels of GAPDH (Santa Cruz Biotechnology, Santa Cruz, CA).

### Cell culture and small interfering RNA transfection

ND7/23 cells (rat Dorsal root ganglia (DRG)/mouse neuroblastoma hybrid) (ND7/23, Lonza, Walkerville, MD) and human schwann cells (hSCs, ScienCell, Carlsbad, CA) were cultured in DMEM (Dulbecco’s Modified Eagle Medium) (Gibco, Thermo Fisher Scientific, Waltham, MA) and schwann cell medium (SCM, ScienCell, Carlsbad, CA), respectively, at 37 °C in a humidified atmosphere containing 5% CO_2_ and 95% air. Passages 4–8 were used in all experiments. For small interfering RNA (siRNA) transfection, ND7/23 and Schwann cells (SCs) were seeded in 6-well plates at a density of 2 × 10^5 cells per well and incubated overnight to reach 60% confluency. The cells were then transfected with 50 nM of target-specific siRNA (*AdipoR1* siRNA: 5’-GGACAACGACUAUCUGCUACATT-3’, *AdipoR2* siRNA: 5’-CCAACUGGAUGGUACACGA-3’) or scrambled siRNA (5’-CCUACGCCACCAAUUUCGU-3’, Bioneer, Daejeon, Korea) using Lipofectamine RNAiMAX (Invitrogen, Carlsbad, CA) with Opti-MEM (Gibco, Thermo Fisher Scientific, Waltham, MA) according to the manufacturer’s instructions. The target-specific siRNAs were designed to knock down the expression of AdipoR1 and AdipoR2. After 5 h of transfection, the medium was replaced with fresh complete medium, and the cells were incubated for an additional 24 h to allow for siRNA uptake and gene silencing. After transfection, SCs were treated with AdipoRon (50 nM; AdipoGen Life Sciences, San Diego, CA), high glucose (35 mmol/L D-glucose; Sigma-Aldrich, St. Louis, MO), and palmitate (0.5 mM; Sigma-Aldrich, St. Louis, MO) for an additional 24 h before harvesting. ND7/23 cells were treated with AdipoRon (50 nM) and high glucose (35 mmol/L D-glucose) for 24 h and with palmitate (0.5 mM) for 3 h before harvesting. Unless otherwise indicated, all treatments were performed in complete medium without serum starvation.

### Immunofluorescence in ND7/23 and Schwann cells

ND7/23 and Schwann cells were fixed in 4% paraformaldehyde and permeabilized with 0.1% Triton X-100. In ND7/23 cells, each monocarboxylate transporter (MCT1, MCT2, and MCT4; Proteintech, USA) was co-stained with neurofilament (NF; Santa Cruz Biotechnology, USA) to assess axonal localization. LDHA and LDHB (Proteintech, USA) were also evaluated using double IF. Autophagy and apoptosis were assessed by staining for LC3 (Sigma-Aldrich, USA) and performing TUNEL assay using the ApopTag Fluorescein In Situ Apoptosis Detection Kit (Millipore, USA), respectively. To evaluate oxidative stress, cells were treated with 5 µM dihydroethidium (DHE; Invitrogen, USA) for 30 min at 37 °C. Mitochondrial superoxide production was assessed using 5 µM MitoSOX Red (Invitrogen, Thermo Fisher Scientific, USA) for 10 min. Co-localization of MitoSOX with MCT1, MCT2, or MCT4 was analyzed by double IF. For intracellular calcium imaging, ND7/23 cells were incubated with 5 µM Fluo-4 AM (Invitrogen, Thermo Fisher Scientific, USA) for 30 min at 37 °C. In Schwann cells, MCT1, MCT2, and MCT4 were co-stained with myelin basic protein (MBP; MyBioSource, USA) to assess their localization relative to the myelin marker. LDHA and LDHB were also analyzed by double IF. In addition, double IF staining for MCT4 and MitoSOX Red was performed to assess mitochondrial oxidative stress. All fluorescent images were obtained using a laser scanning confocal microscope system (Carl Zeiss LSM 900, Germany). Additionally, to assess cellular metabolism, lactate (Cell Biolabs, Inc., San Diego, CA), ATP (Abcam, Cambridge, UK), and adenosine monophosphate (AMP; Abcam, Cambridge, UK) levels were measured in cell lysates from both ND7/23 and Schwann cells using commercial kits, following the manufacturers’ protocols.

### Data analysis

Results are presented as mean ± standard deviation (SD). Group differences were analyzed using two-way ANOVA with subsequent Bonferroni correction (SPSS v. 11.5, IBM, Armonk, NY, USA). A *p*-value of less than 0.05 was considered statistically significant.

## Results

### Biochemical and physical characteristics of db/db and db/m mice with or without Adiporon treatment

At the end of the study, the body weights of *db/db* mice were significantly greater than those of *db/m* mice in both the AdipoRon treatment and control groups. AdipoRon treatment significantly reduced the elevated HOMA-IR index in *db/db* mice. This was accompanied by a marked decrease in serum insulin levels in *db/db* mice. Total cholesterol levels were elevated in *db/db* mice in both the AdipoRon treatment and control groups. AdipoRon treatment significantly reduced triglyceride (TG) and free fatty acid (FFA) levels in *db/db* mice. Serum adiponectin levels were significantly decreased in *db/db* mice in both treatment and control groups. The elevated 24-hour urinary 8-OH-dG and isoprostane levels in *db/db* mice were significantly reduced with AdipoRon treatment. AdipoRon treatment did not alter HbA1c and adiponectin concentrations in either *db/db* or *db/m* mice (Table 1).

**Table 1:** Biochemical and physical characteristics of all study groups

	db/m cont	db/m + AdipoR	db/db cont	db/db + AdipoR
**Body weight (gm)**	36.1 ± 1.9	37.0 ± 2.0	53.3 ± 4.9^#^	51.6 ± 4.7^#^
**HbA1c (%)**	4,0 ± 0.1	3.9 ± 0.2	10.7 ± 0.9^#^	11.4 ± 1.0^#^
**HOMA**_**IR**_	0.06 ± 0.01	0.06 ± 0.01	2.43 ± 0.25^#^	0.21 ± 0.04^**^
**Serum total cholesterol (mmol/L)**	2.72 ± 0.27	2.62 ± 0.35	3.60 ± 0.37^**^	3.36 ± 0.39^*^
**Serum triglycerides (mmol/L)**	1,22 ± 0.21	1.32 ± 0.21	2.10 ± 0.37^#^	1.66 ± 0.16^*^
**Serum Free fatty acid (mmol/L)**	0.62 ± 0.09	0.67 ± 0.13	1.42 ± 0.11^#^	1.11 ± 0.11^**^
**Serum adiponection (ng/ml)**	9919 ± 1138	10,999 ± 1138	4827 ± 472^#^	4903 ± 556^#,**^
**24-hr urinary 8-OH-dG (ng)**	39.2 ± 12.1	35.3 ± 16.3	187.5 ± 52.8^#^	49.3 ± 14.4^#,**^
**24-hr urinary isoprotane (ng)**	5.3 ± 1.2	4.6 ± 1.8	47.4 ± 11.2^#^	26.8 ± 5.8^#,**^

Abbreviations; HbA1c, Hemoglobin A1c; HOMA-IR, Homeostasis Model Assessment of Insulin Resistance; 8-OH-dG, 8-Hydroxy-2’-deoxyguanosin ^**^P < 0.01, ^#^*P* < 0.001, compared with other groups

### Changes in sciatic nerve function and morphology in db/db and db/m mice with or without AdipoRon treatment

**Fig. 1:**
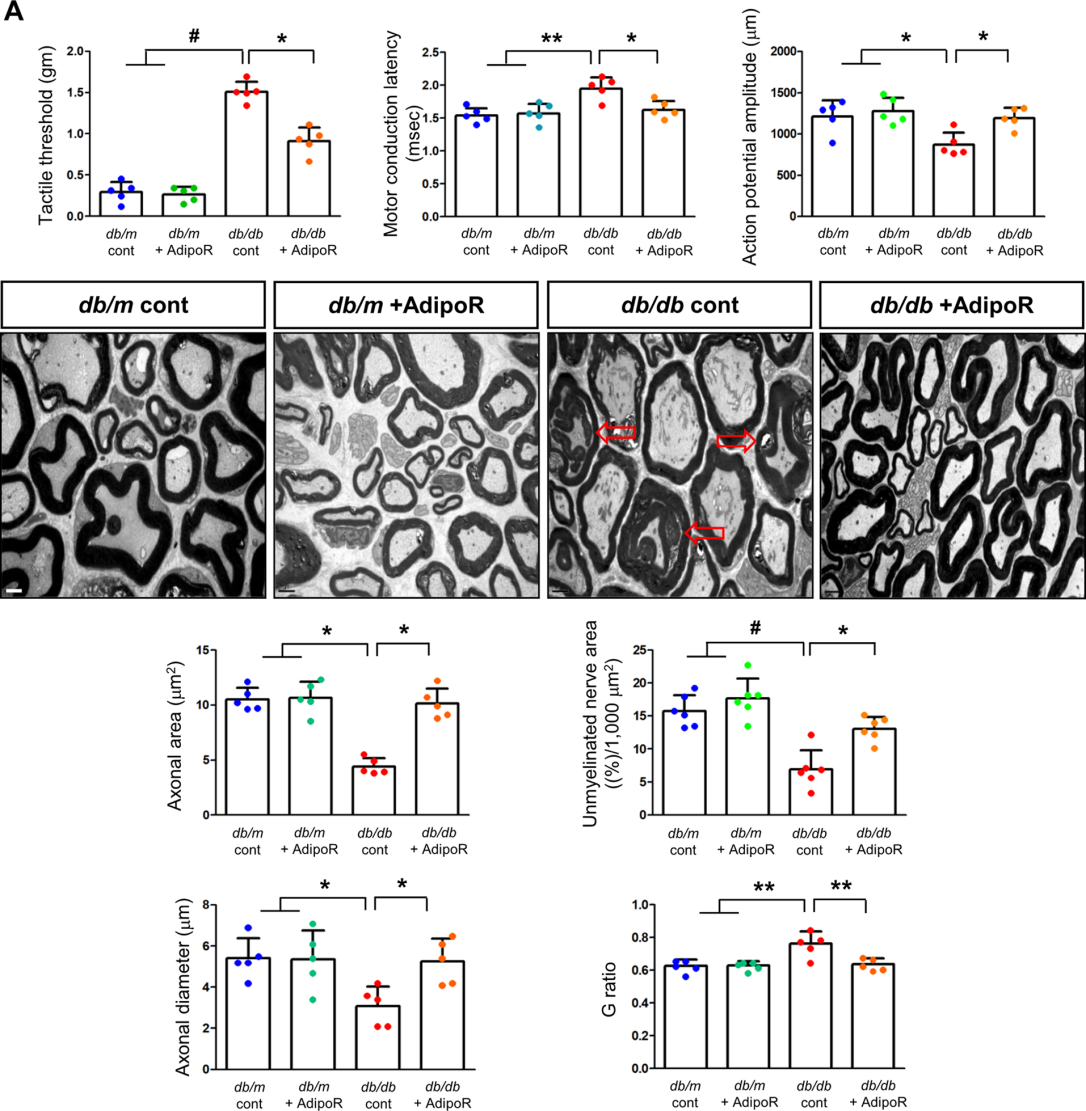

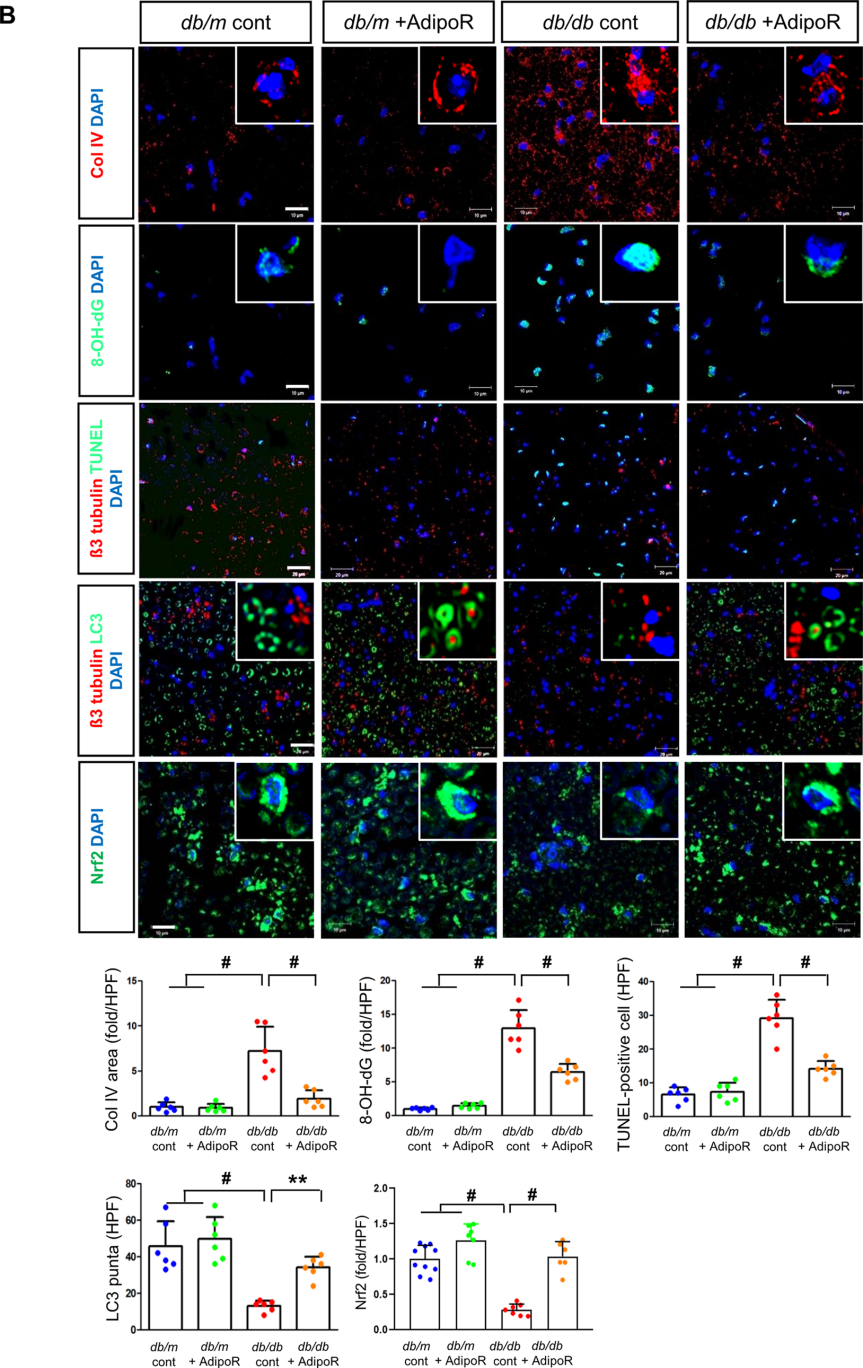

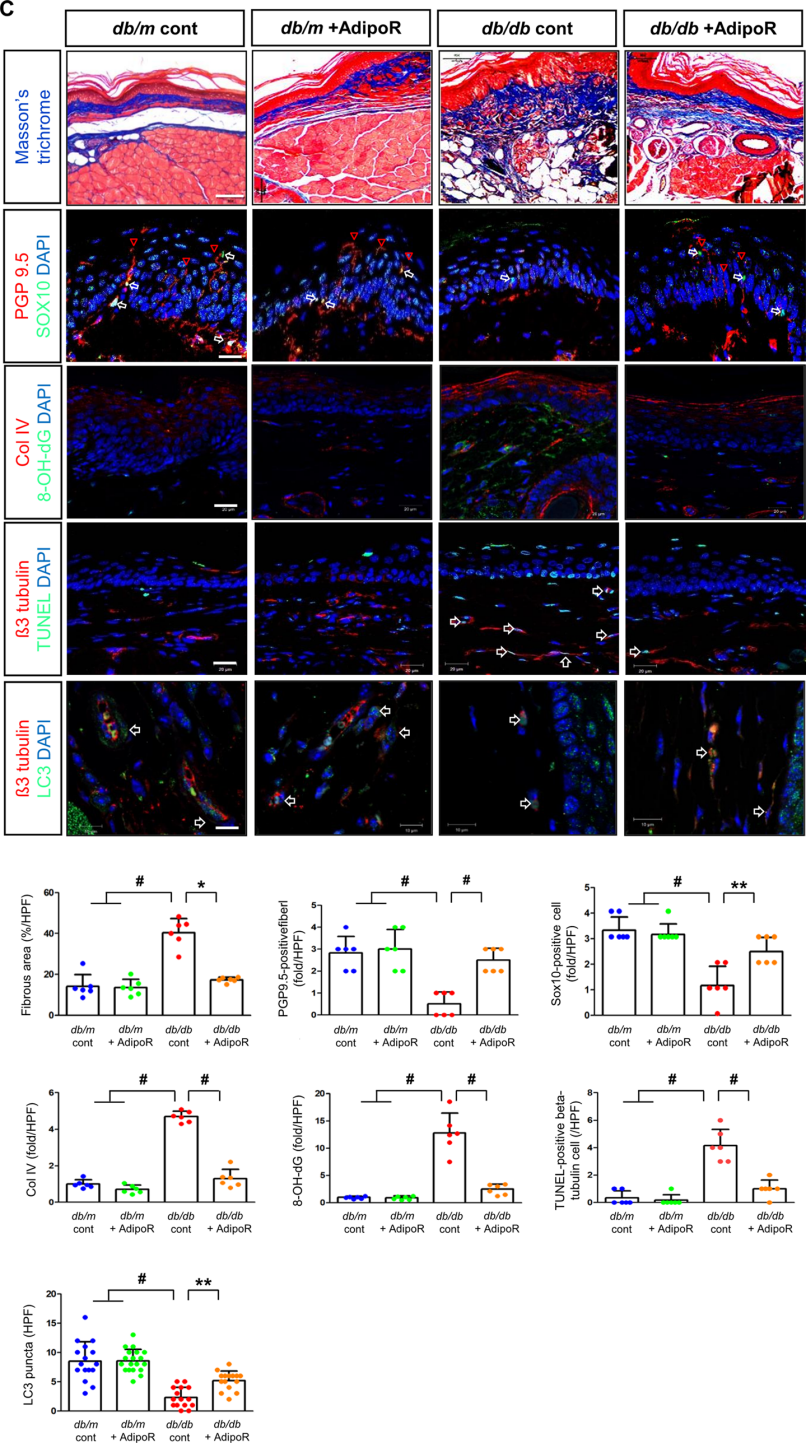
Effects of AdipoRon on nerve function and morphology in diabetic *db/db* and non-diabetic *db/m* mice. (**A**) Tactile thresholds, motor nerve conduction latency (MNCL), and action potential amplitude, along with electron microscopy results showing axonal area, unmyelinated nerve area, axonal diameter, and G-ratio in the sciatic nerve of diabetic *db/db* and non-diabetic *db/m* mice with or without AdipoRon treatment, are shown with quantitative analyses (*n* = 6). Red arrows indicate aberrant structures such as redundant myelin folding, infolding of the myelin sheaths, and degenerated axons (scale bars: 2 μm). (**B**) Immunofluorescence (IF) staining of collagen IV (red, scale bars: 10 μm), 8-OH-dG (green, 10 μm), and total Nrf2 (green, 10 μm) was performed individually. Double IF staining was performed for β3-tubulin (red, 20 μm) with either TUNEL or LC3 (green, 20 μm) to confirm the neuronal localization of apoptotic and autophagic signals. DAPI (blue) was used for nuclear counterstaining, and representative images with their enlarged views from the rectangular areas and corresponding quantitative analyses are shown (*n* = 6). (**C**) Additional experiments in paw skin included Masson’s Trichrome (MT) staining (scale bars: 100 μm) to assess fibrosis. Immunofluorescence (IF) staining included the following double combinations: SOX10 (green, Schwann cell marker, indicated by white open arrows) and PGP9.5 (red, nerve fiber marker, indicated by red open arrowheads) (scale bars: 20 μm); collagen IV (red) and 8-OH-dG (green) (scale bars: 20 μm); and β3-tubulin (red, mature neuron marker) with either TUNEL or LC3 (green**)** (scale bars: 10 μm). DAPI (blue) was used for nuclear counterstaining, and representative images with corresponding analyses are shown (*n* = 6). All analyses were performed in *db/db* and *db/m* mice with or without AdipoRon treatment. Data are presented as the mean ± standard deviation (SD). *P < 0.05, **P < 0.01, and #*P* < 0.001 compared with other groups

Tactile response thresholds and motor nerve conduction latency (MNCL) were higher in *db/db* control mice than in *db/m* control mice at the end of the 20-week study. AdipoRon treatment significantly improved the tactile response threshold and MNCL in *db/db* mice to levels similar to those observed in *db/m* mice. This improvement was consistent with an increase in action potential amplitude in *db/db* mice treated with AdipoRon. In contrast, there were no changes in tactile response, sciatic motor conduction latency, or action potential amplitude in *db/m* mice, regardless of AdipoRon treatment (Fig. 1A).

Axonal area, unmyelinated fiber area, and SC degeneration in the sciatic nerves of *db/m* and *db/db* mice were assessed using electron microscopy at 20 weeks. The *db/db* control mice showed marked decreases in axonal area and diameter, which were restored to levels comparable to those of *db/m* control mice treated with AdipoRon. The G-ratio, a quantitative measure of relative myelin thickness, was elevated in *db/db* control mice but decreased to levels similar to *db/m* control mice following AdipoRon treatment. Additionally, the reduced area of unmyelinated nerve fibers observed in *db/db* control mice was restored by AdipoRon treatment. The prominent redundant myelin folding, infolding of the myelin sheaths, and degenerated axons noted in *db/db* control mice were not present in the AdipoRon treatment group (Fig. 1A).

### Changes in sciatic nerve apoptosis, autophagy activity, cellular differentiation, fibrosis, and metabolic activity in db/db and db/m mice with or without AdipoRon treatment

The increased expression of collagen IV and 8-OH-dG in *db/db* mice was ameliorated by AdipoRon treatment. β3-tubulin, a marker of mature neurons, showed increased staining with TUNEL and decreased staining with LC3 in *db/db* mice, indicating increased apoptosis and decreased autophagic activity. These effects were ameliorated by AdipoRon treatment, suggesting decreased apoptosis and increased autophagy in sciatic nerve (Fig. 1B). Moreover, after AdipoRon treatment, Nrf2 levels, which were decreased in both the cytoplasm and nucleus of neurons in *db/db* mice, were increased (Fig. 1B). This suggests that the activation of the Nrf2 antioxidant pathway led to a reduction in oxidative stress. Masson’s trichrome staining of the paw skin revealed disarranged bundles of nerve fibers in *db/db* mice, which was ameliorated by AdipoRon treatment. The increased expression of β3-tubulin co-staining with TUNEL-positive cells in *db/db* mice was significantly decreased with AdipoRon treatment (Fig. 1C).

PGP9.5, a marker for peripheral nerve fibers, functions as a tissue-specific ubiquitin carboxyl-terminal hydrolase isoenzyme. SOX10, a transcription factor involved in the development and function of neural crest cells, which give rise to various cell types in the PNS, showed decreased expression upon co-staining with PGP9.5 in *db/db* mice; this increased with AdipoRon treatment. The increased expression of Col-IV and 8-OH-dG in *db/db* mice were decreased with AdipoRon treatment, while the decreased expression of β3-tubulin and LC3 in *db/db* mice was increased with AdipoRon treatment (Fig. 1C).

### Changes in sciatic nerve expression of adiponectin receptors and associated target molecules in db/db and db/m mice with or without AdipoRon treatment

**Fig. 2:**
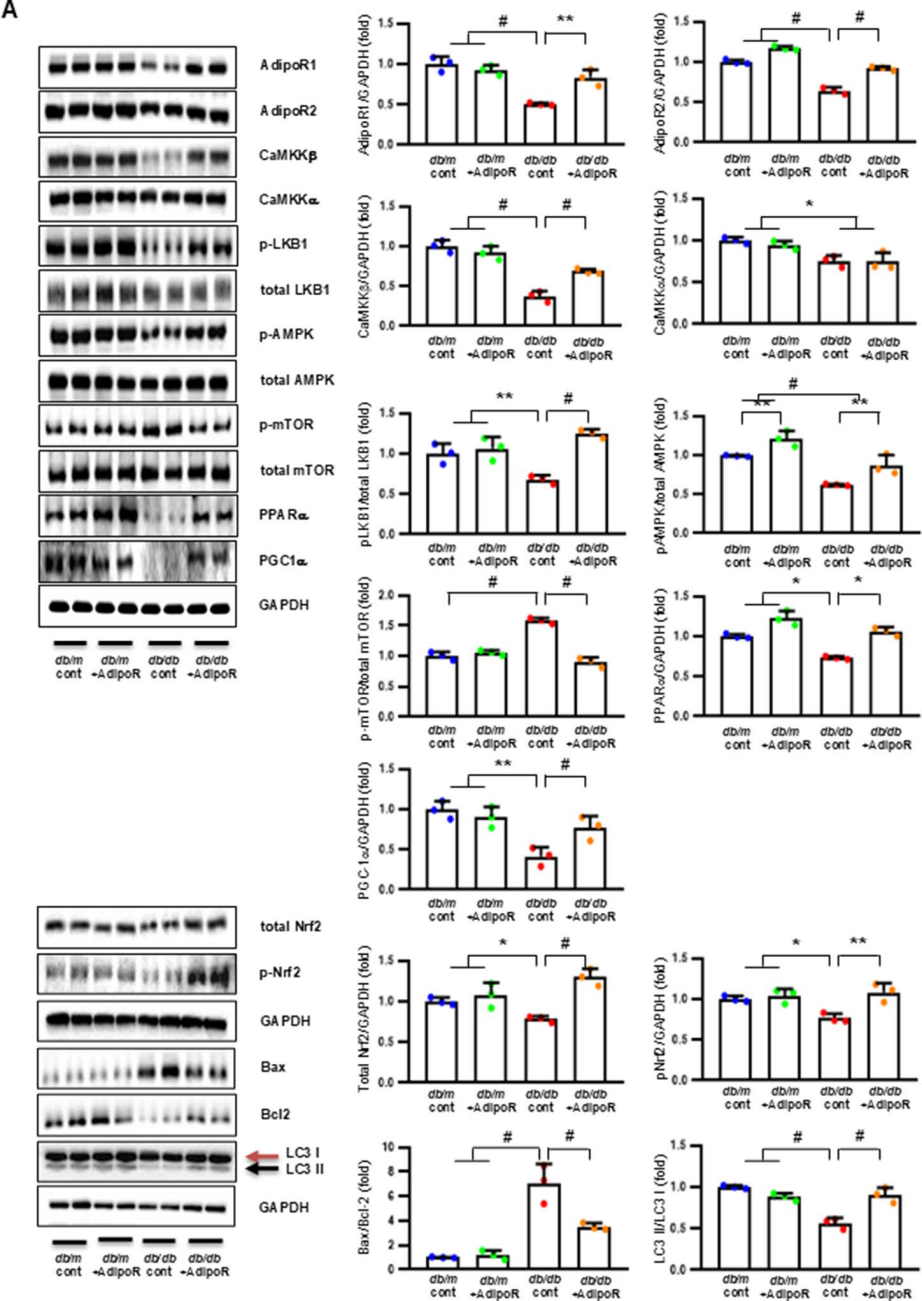

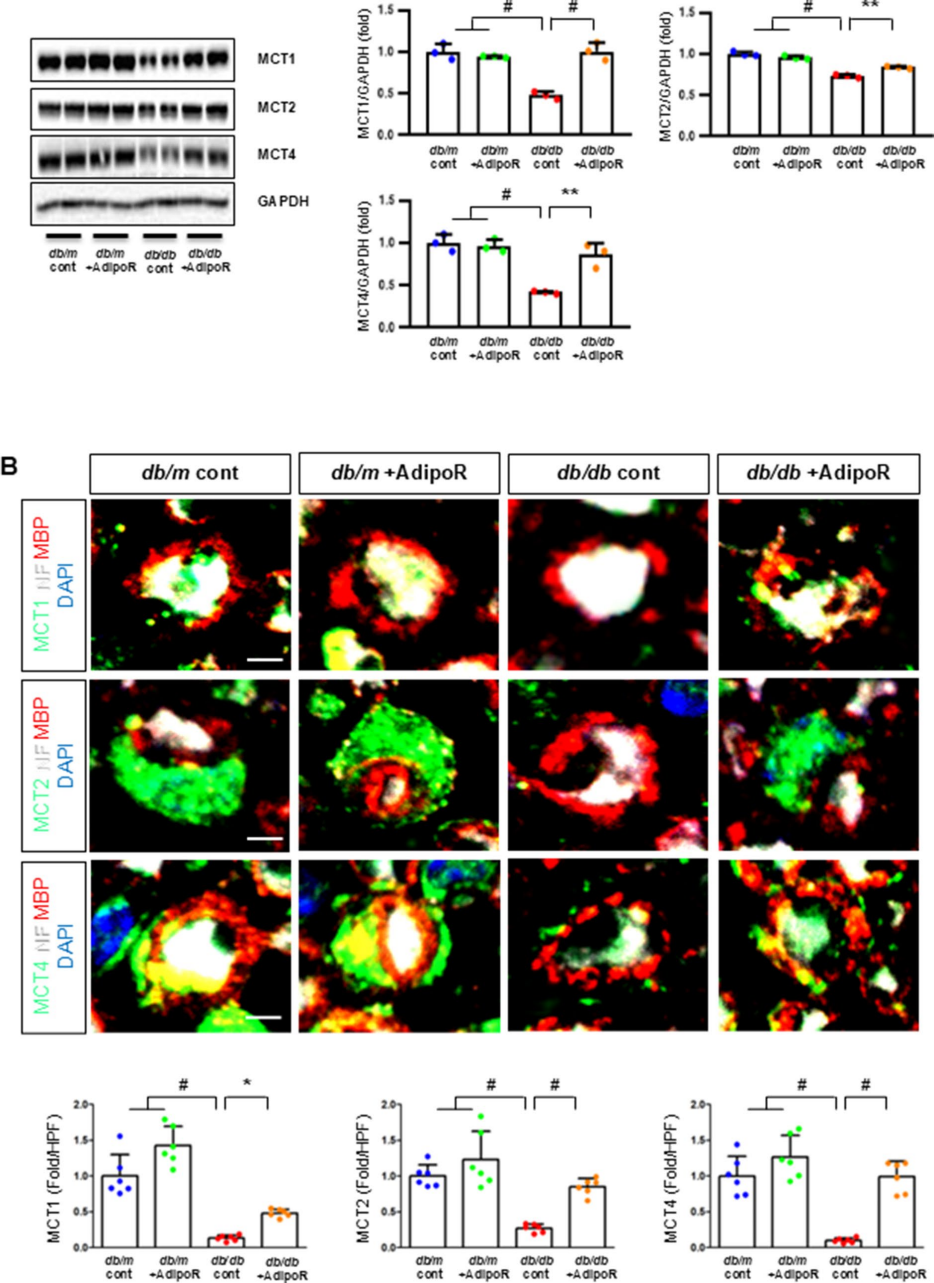

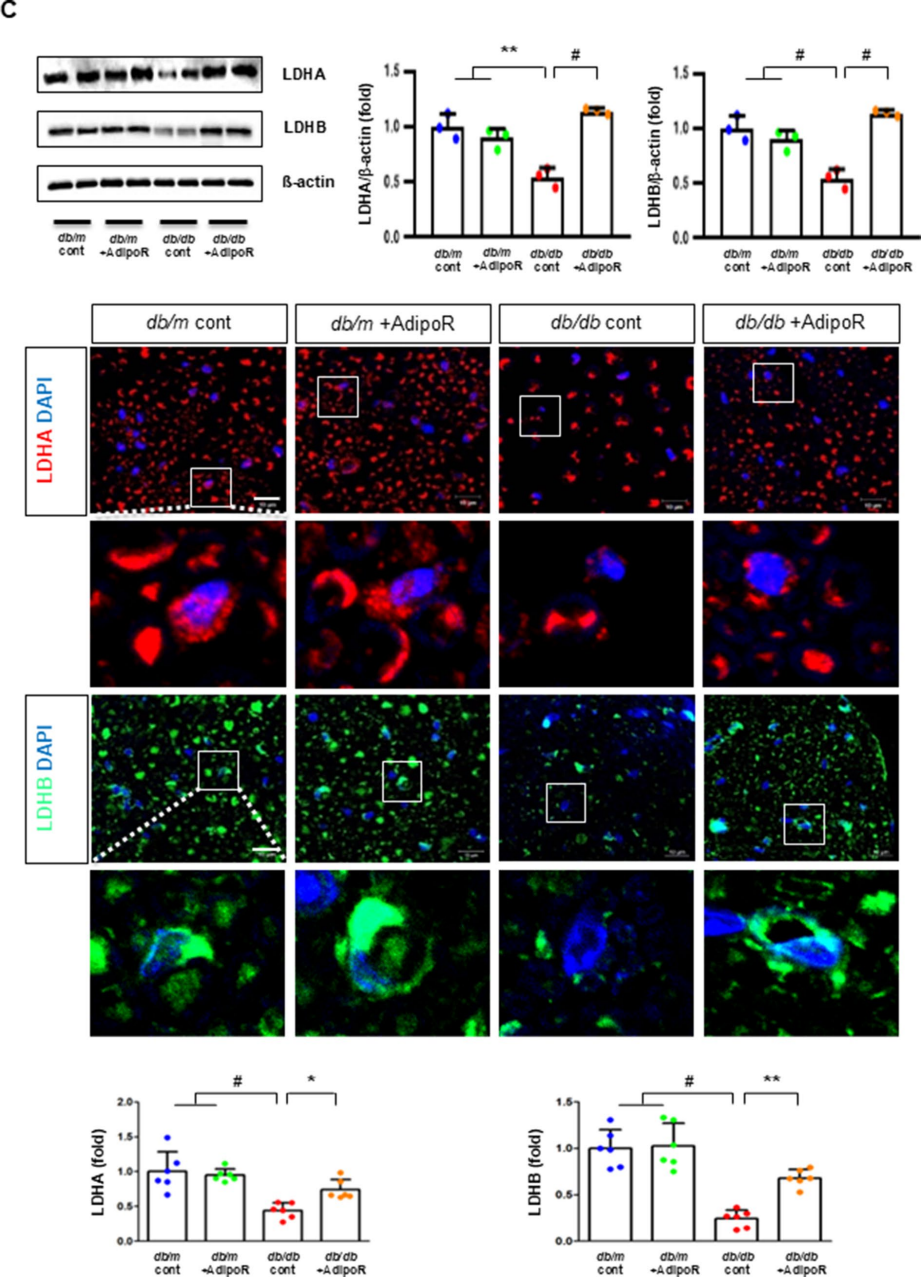

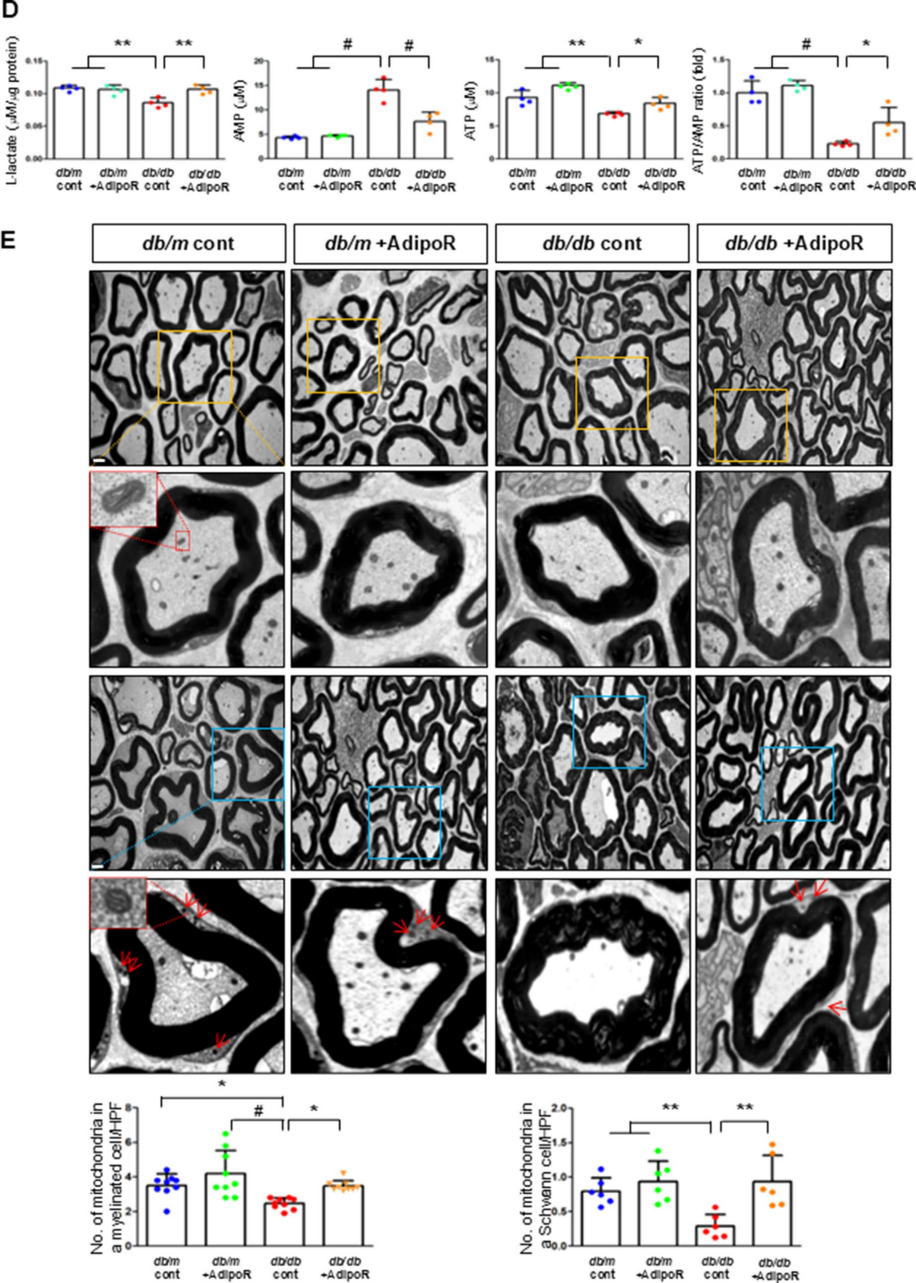
Changes in the expressions of adiponectin receptors and associated downstream signaling pathways in the sciatic nerve of diabetic *db/db* and non-diabetic *db/m* mice with or without AdipoRon treatment. (**A**) Representative western blot images of AdipoR1, AdipoR2, CaMKKα and β, pLKB1/total LKB1, pAMPK/total AMPK, p-mTOR/total mTOR, PPARα, PGC-1α, p-Nrf2/total Nrf2, MCT1, MCT2, MCT4, GAPDH, and Bax/Bcl-2 and LC3-II/LC3-I ratios in the sciatic nerve are shown with quantitative analyses (*n* = 3). (**B**) Representative images of triple IF staining for MCT1, MCT2, and MCT4 (green), NF (white, axonal marker), and MBP (red, myelin marker) in the sciatic nerve of *db/db* and *db/m* mice with or without AdipoRon are shown with corresponding quantitative analyses (*n* = 6). (**C**) Expression levels of LDHA and LDHB in the sciatic nerve after AdipoRon treatment, evaluated by western blot (*n* = 3) and IF analysis and their enlarged images from the rectangular areas, respectively, are shown with quantitative analyses (*n* = 6). (**D**) Quantitative measurements of lactate, AMP, and ATP levels, including the ATP/AMP ratio, in the sciatic nerve of *db/db* mice with or without AdipoRon treatment (*n* = 4). (E) Representative electron microscopy images of the sciatic nerve (x 5,000, scale bars: 2 μm) in *db/db* and *db/m* mice with or without AdipoRon treatment and their enlarged images from the rectangular areas, respectively. Quantitative analyses of mitochondrial number in myelinated axons (*n* = 10) and Schwann cells (*n* = 6). Data are presented as the mean ± SD. *P < 0.05, **P < 0.01, and #*P* < 0.001 compared with other groups. Scale bars in (B) represent 2 μm, and in (**C**), 10 μm

Decreased expression of both AdipoR1 and AdipoR2 in *db/db* mice significantly increased with AdipoRon treatment. Similarly, decreased levels of CaMKKβ, phospho-LKB1 (pLKB1), and pAMPK in *db/db* mice were elevated with AdipoRon treatment; the increase in pAMPK expression was consistent with the upregulation of its downstream target molecules with AdipoRon, including PPARα, PGC-1α, and pNrf2 and total Nrf2, and the downregulation of p-mTOR. Additionally, the increased Bax/Bcl-2 and decreased LC3-II/LC3-I ratio in *db/db* mice were ameliorated by AdipoRon treatment. Decreased expression of MCT1 and MCT2 in *db/db* mice, especially in the periphery of myelin sheaths and axons, and MCT4, particularly in the perinuclear region of neurons, was restored with AdipoRon treatment (Fig. 2A). To evaluate the expression and localization of MCT isoforms in sciatic nerve, we performed triple immunofluorescence staining using NF, MBP, and each of MCT1, MCT2, or MCT4. In *db/m* mice, MCT1 showed strong co-localization with NF but not with MBP, indicating predominant axonal localization. MCT2 was primarily co-localized with MBP, indicating localization within the Schwann cell myelin sheath. MCT4 displayed dual localization, co-localizing with both NF and MBP, suggesting distribution in both axonal and myelin compartments. These expression levels and localization patterns were markedly reduced in *db/db* mice but were significantly restored with AdipoRon treatment (Fig. 2B). In the same line, the decreased LDHA and LDHB expression of in *db/db* mice were significantly increased after AdipoRon treatment as shown by IF and WB analysis (Fig. 2C). Quantitative measurements of lactate, AMP, and ATP in the sciatic nerve showed that the decreased lactate and ATP levels in *db/db* mice were significantly increased via AdipoRon treatment, along with a collective increase in the ATP/AMP ratio (Fig. 2D). Electron microscopy revealed that the number of mitochondria in both myelinated cells and SCs of *db/db* mice significantly increased following AdipoRon treatment, reversing the observed decrease in mitochondria in these cells (Fig. 2E).

### AdipoRon modulates AdipoR1/AdipoR2 expressions via the LKB, CaMKK, pAMPK, and PPARα PGC-1α signaling pathway in HSCs and ND7/23 cells

**Fig. 3:**
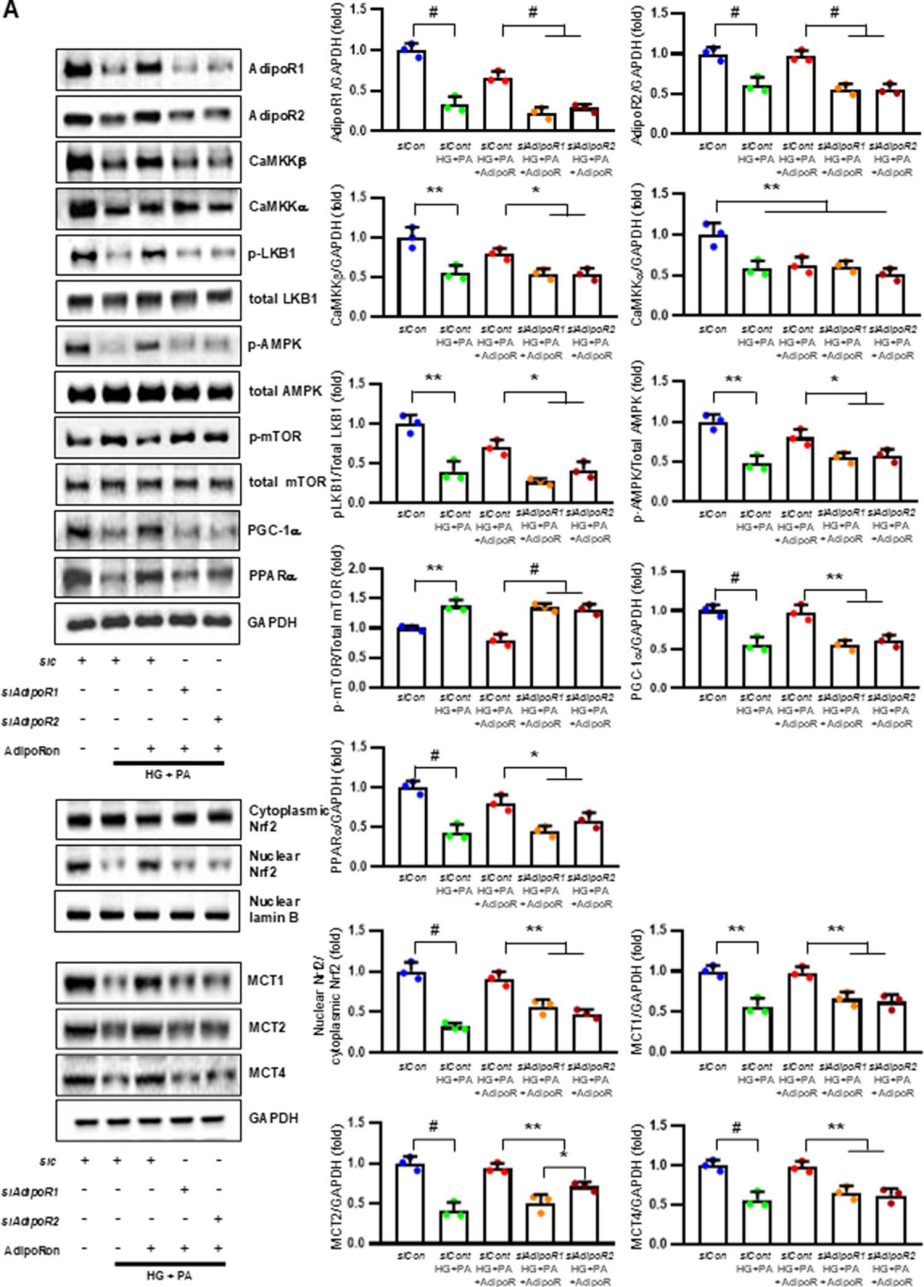

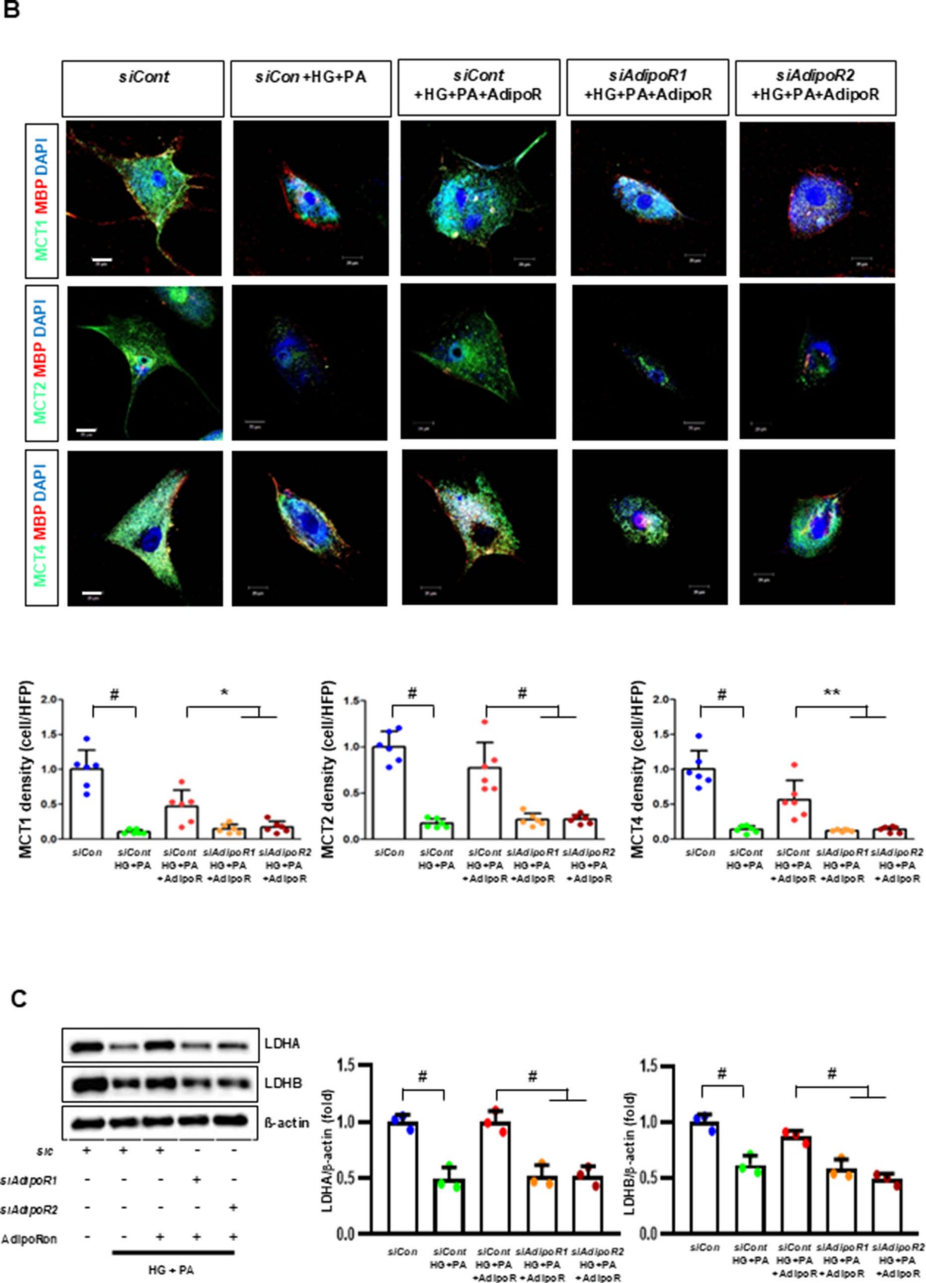

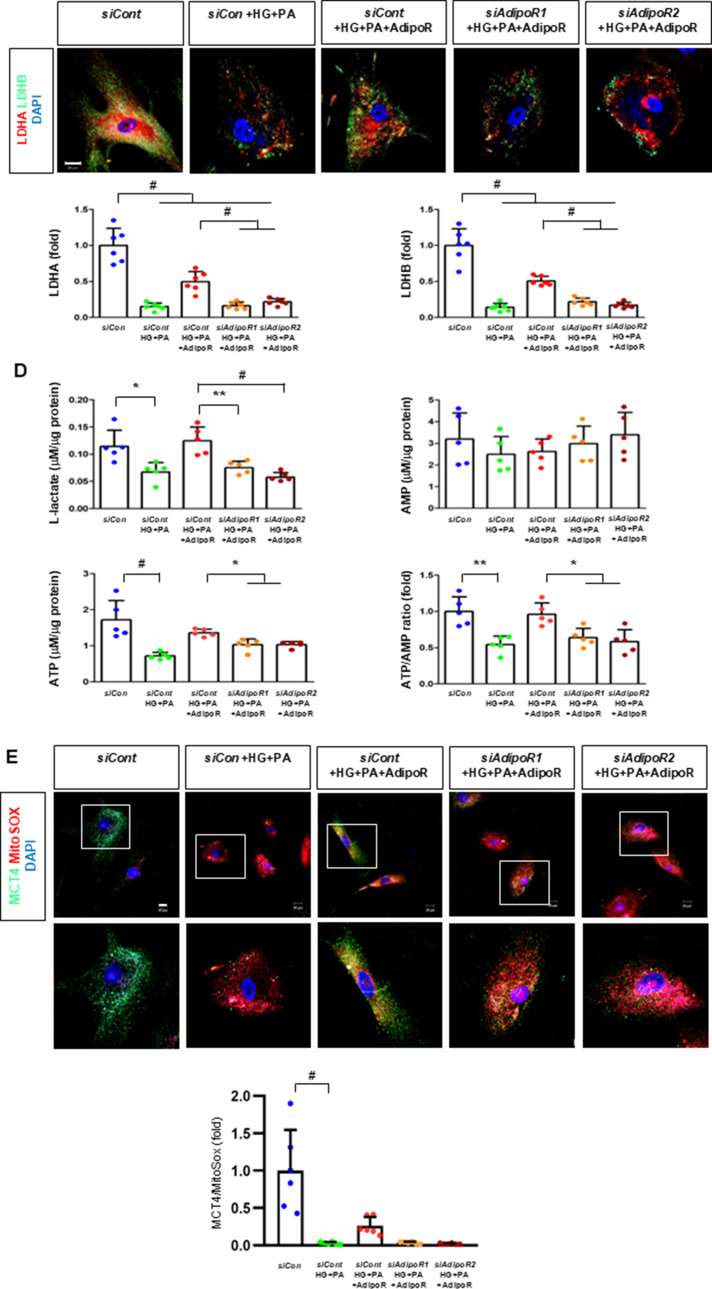
AdipoRon modulates AdipoR1 and AdipoR2 expression in human Schwann cells (HSCs). Human Schwann cells (HSCs) were transfected with *adipoR1* or *adipoR2* siRNA and cultured in high glucose and palmitate medium, with or without AdipoRon treatment. (**A**) Representative western blot images showing the expression of AdipoR1, AdipoR2, CaMKKα and β, pLKB1/total LKB1, pAMPK/total AMPK, p-mTOR/total mTOR, PGC-1α, PPARα, MCT1, MCT2, MCT4, GAPDH, cytoplasmic Nrf2, nuclear Nrf2, and lamin B. Quantitative analyses of protein expression levels are shown (*n* = 3). (**B**) Representative images of double IF staining for MCT1, MCT2, and MCT4 (green) with MBP (red, myelin marker) are shown with corresponding quantitative analyses (*n* = 6). (**C**) Representative western blot (*n* = 3) and IF images (*n* = 6) for LDHA and LDHB expression are shown with quantitative analyses. (**D**) Quantitative measurements of intracellular lactate, AMP, and ATP levels, including the ATP/AMP ratio, are shown (*n* = 5). (**E**) Representative double immunofluorescence staining images of MCT4 (green) and MitoSOX (red) in HSCs under different conditions (*n* = 6). Data are presented as the mean ± SD. *P < 0.05, **P < 0.01, and #*P* < 0.001, compared with other groups. Scale bars in (B, C and E) represent 20 μm

**Fig. 4:**
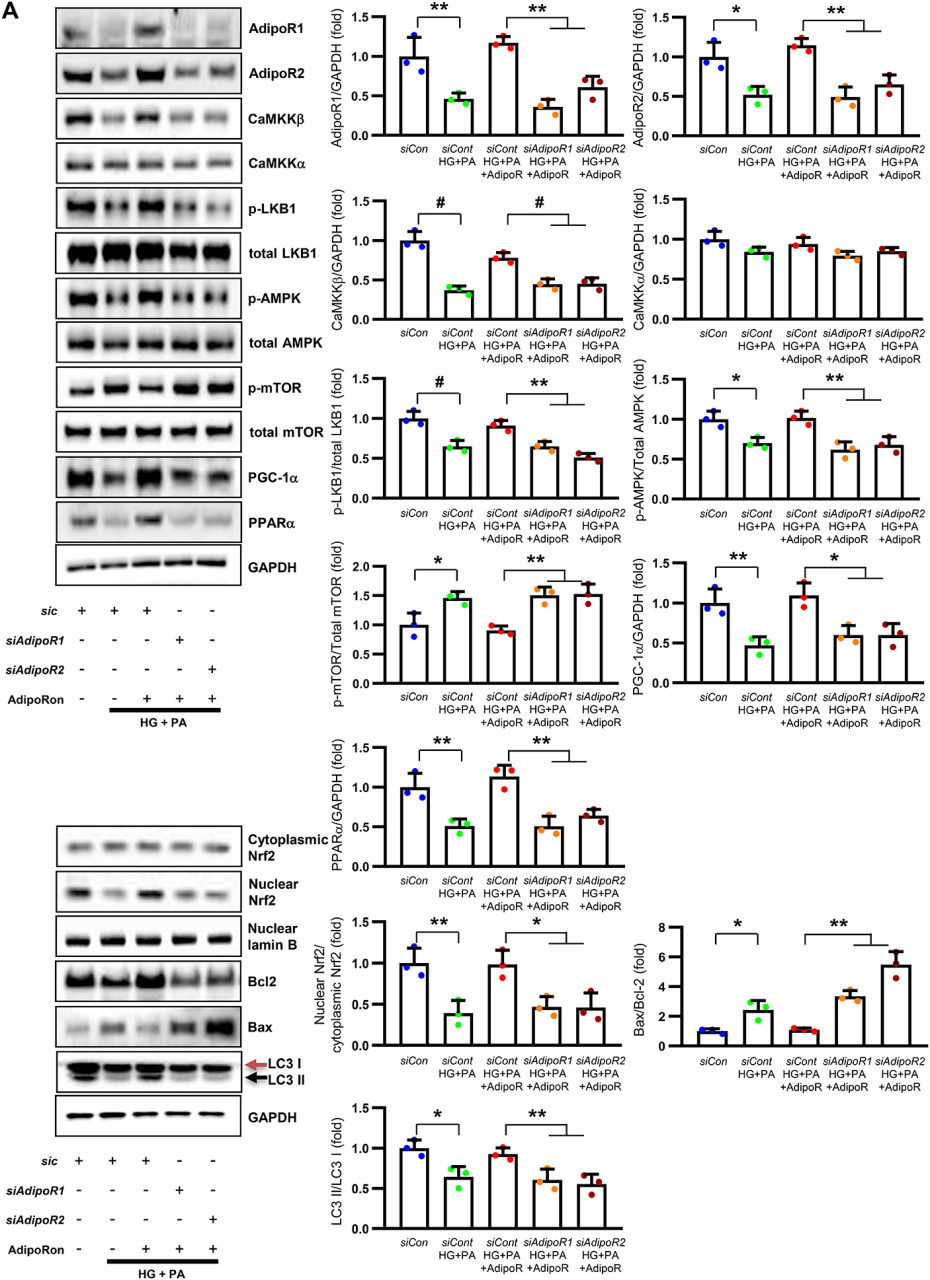

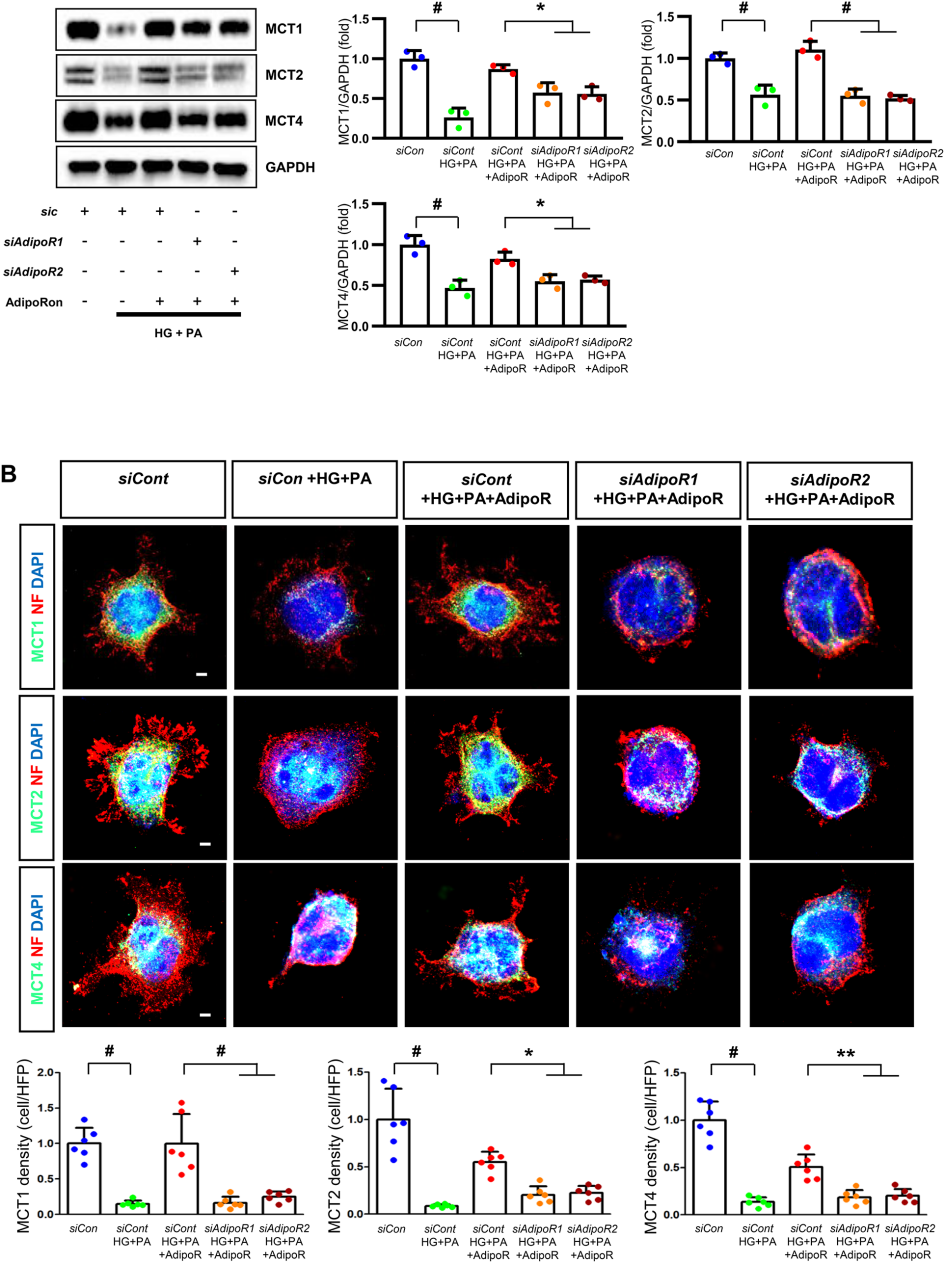

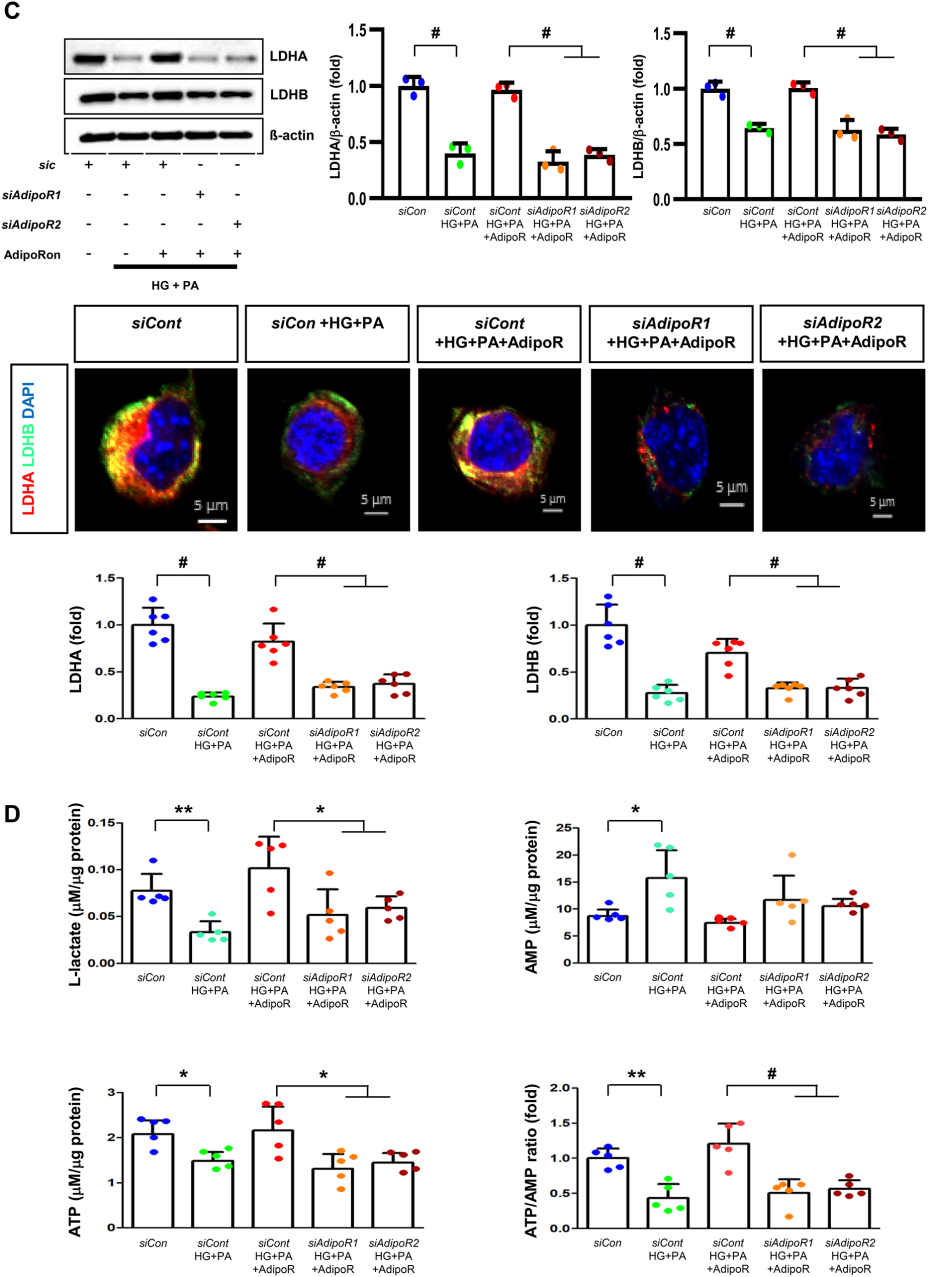

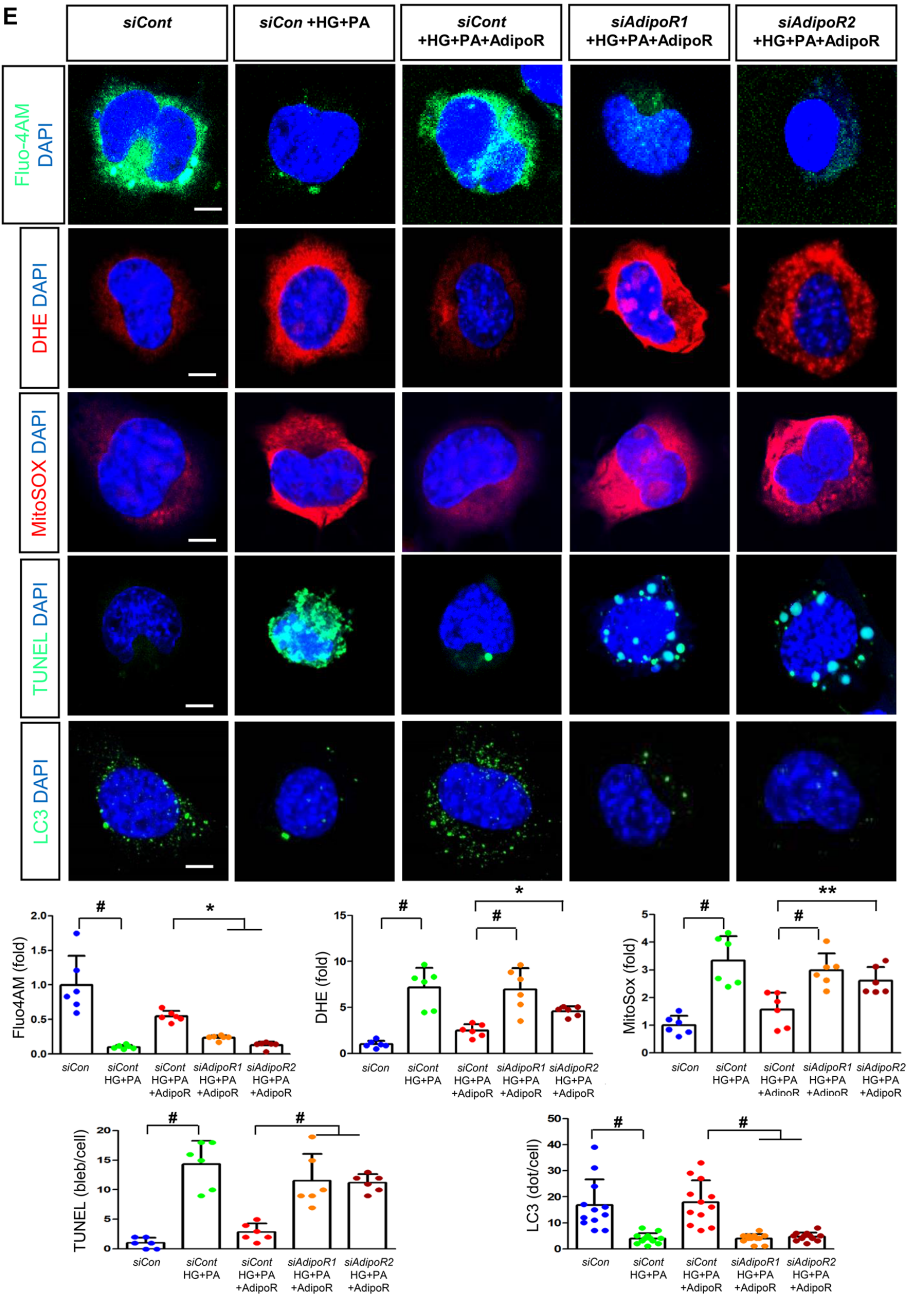

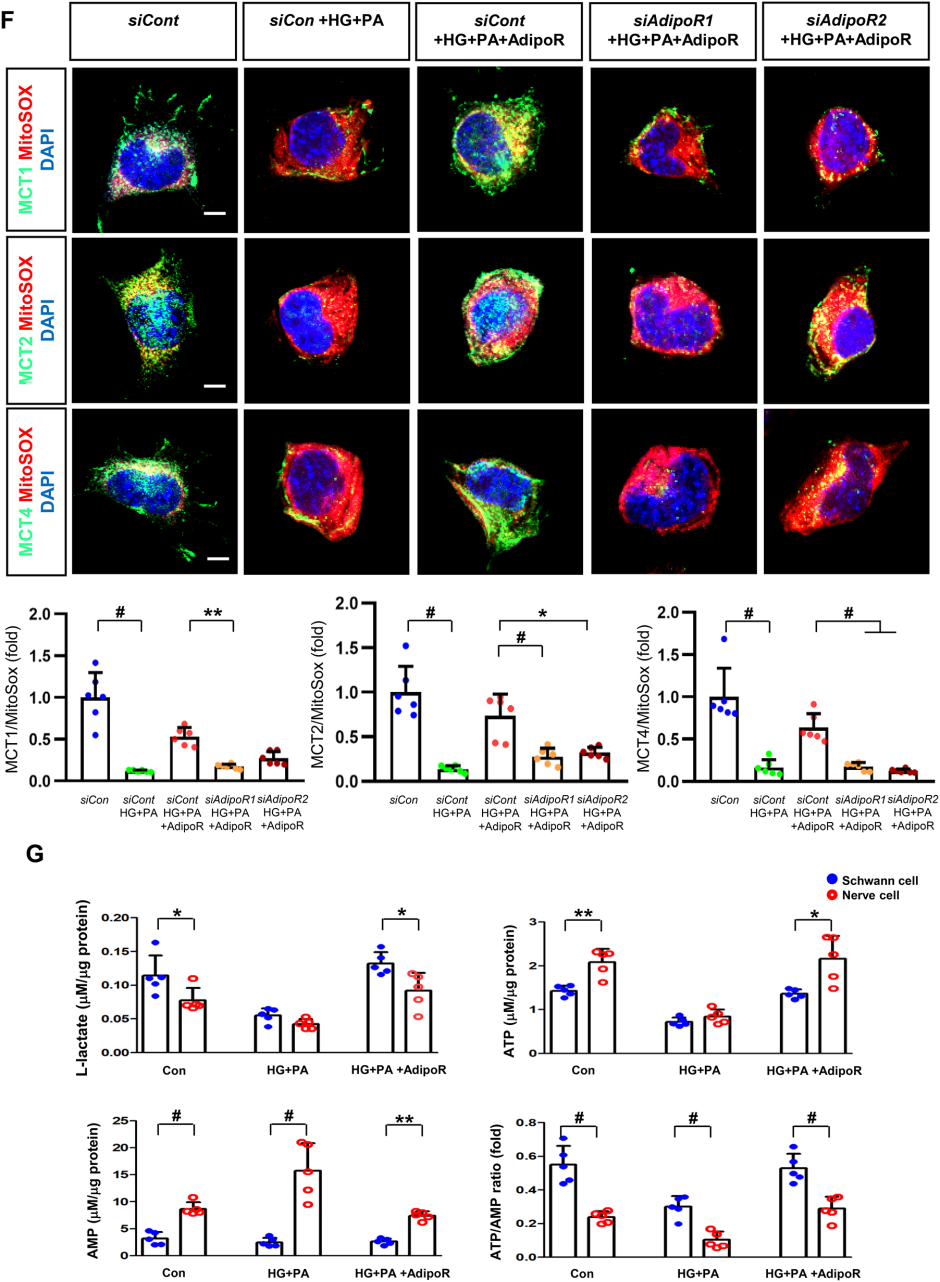
Effect of AdipoRon on signaling pathways in ND7/23 cells (murine DRG neuronal cell). ND7/23 cells were transfected with *adipoR1* or *adipoR2* siRNA and cultured in low- or high-glucose (HG) and palmitate (PA) medium, with or without AdipoRon treatment. (**A**) Representative western blot images showing the expression of AdipoR1, AdipoR2, CaMKKα and β, pLKB1/total LKB1, pAMPK/total AMPK, p-mTOR/total mTOR, PGC-1α, PPARα, Bax/Bcl-2, LC3II/LC3I ratios, MCT1, MCT2, MCT4, GAPDH, cytoplasmic Nrf2, nuclear Nrf2, and lamin (**B**). Quantitative analyses of protein expression levels in each group are shown (*n* = 3). (B) Representative images of double IF staining for MCT1, MCT2, and MCT4 (green) with NF (white, axonal marker) are shown with corresponding quantitative analyses (*n* = 6). (**C**) Representative western blot (*n* = 3) and double IF images (*n* = 6) for LDHA (red) and LDHB (green) expression, with quantitative analyses. (**D**) Quantitative measurements of intracellular lactate, AMP, and ATP levels, and ATP/AMP ratio (*n* = 5). (**E**) Representative sections stained with Fluo-4AM (green, intracellular Ca^++^), DHE (red, cellular oxidative stress), MitoSox (red, mitochondrial oxidative stress), TUNEL (green, apoptosis), and LC3 (green, autophagy). Quantitative analyses of positive areas and density are shown for each group. Data are presented as the mean ± SD (*n* = 6). (**F**) Representative double IF staining images of MCT1, MCT2, MCT4 (green) and MitoSOX (red) in ND7/23 cells under different conditions (*n* = 6). **(G) **Quantitative measurements of intracellular lactate, ATP and AMP concentrations and ATP/AMP ratios in SCs and ND7/23 cells in low glucose (LG), high glucose and palmitate (HG + PA), and high glucose and palmitate treated with adiopoRon (HG + PA + adipoR), respectively (*n* = 5). *P < 0.05, **P < 0.01, and #*P* < 0.001, compared with other groups. Scale bars in (B, C, E and F) represent 5 μm

HSCs (Fig. 3) and ND7/23 cells (Fig. 4) cultured in a high glucose and palmitate medium, showed decreased expression of both AdipoR1 and AdipoR2, as well as CaMKKβ, pLKB1, pAMPK, PPARα and PGC-1α in western blot analysis. In contrast, p-mTOR was upregulated. Additionally, they showed decreased expression of intracellular MCT1, MCT2, MCT4, LDHA, and LDHB in WB and IF analysis, and low levels of intracellular lactate, ATP, and ATP/AMP compared to those cultured in low glucose medium. These changes resulted in an increased Bax/Bcl-2 ratio and decreased LC3-II/LC3-I and nuclear/cytoplasmic Nrf2 ratios in ND7/23 cells, indicating increased susceptibility to apoptosis and oxidative stress, as well as compromised autophagy (Figs. 3 and 4).

HSCs cultured in high glucose and palmitate media were transfected with small interfering RNAs (siRNAs) targeting the genes encoding *AdipoR1* and *AdipoR2* to determine which AdipoRs were responsible for activating downstream effectors. AdipoRon treatment did not increase the expression of AdipoR1 and AdipoR2 in hSCs transfected with either *AdipoR1* or *AdipoR2* siRNA; additionally, the expression of CaMKKβ did not increase with AdipoRon treatment in siRNA-treated cells. Although the expression of pLKB1 and pAMPK did not increase with AdipoRon treatment, the expression of p-mTOR increased in hSCs transfected with *AdipoR1/R2* siRNA. Additionally, the expression of PGC-1α, PPARα, MCT1, MCT2, and MCT4 did not increase with AdipoRon treatment in HSCs transfected with AdipoR1/R2 siRNA (Fig. 3A). Immunofluorescence staining showed that AdipoRon did not increase the expression of MCT1, MCT2, and MCT4, which were co-stained with MBP in hSCs transfected with *AdipoR1/R2* siRNAs (Fig. 3B). Measurement of intracellular LDHA, LDHB, AMP, and ATP levels revealed that AdipoRon increased LDHA, LDHB, and AMP levels as well as decreased lactate and ATP levels, collectively reducing LAHA, LDHB, and ATP/AMP ratio in hSCs transfected with *AdipoR1/R2* siRNA (Fig. 3C, D). Additionally, immunofluorescence staining was performed in SCs transfected with *AdipoR1* and *AdipoR2* siRNAs. AdipoRon treatment did not increase MCT4 expression or reduce mitochondrial oxidative stress, as indicated by MitoSOX staining compared with those of the control SCs (Fig. 3E). These findings suggest that AdipoRon’s protective metabolic effects on hSCs were mediated by both adipoR1 and adipoR2 together and that MCT4 expression after AdipoRon treatment was associated with mitochondrial oxidative stress.

ND7/23 cells cultured in high glucose and palmitate media were transfected with siRNAs targeting genes encoding *AdipoR1* and *AdipoR2* (Fig. 4). AdipoRon treatment did not increase AdipoR1 and AdipoR2 expression in transfected ND7/23 cells; similarly, the expression of CaMKKβ, pLKB1, or pAMPK did not increase with AdipoRon treatment in these transfected cells. While the expression of p-mTOR increased, the expression of PGC1-α decreased with AdipoRon in ND7/23 cells transfected with *AdipoR1/R2* siRNA. The expression levels of PPARα, MCT1, MCT2, MCT4, and both cytoplasmic and nuclear Nrf2 did not increase with AdipoRon treatment in ND7/23 cells transfected with *AdipoR1/R2* siRNA. Additionally, AdipoRon treatment did not decrease the Bax/Bcl-2 ratio or increase the LC3-II/LC3-I ratio in these transfected cells (Fig. 4A). Double Immunofluorescence staining showed that AdipoRon treatment did not increase the expression of MCT1, MCT2, or MCT4, which were co-stained with NF in ND7/23 cells transfected with *AdipoR1/R2* siRNAs (Fig. 4B). The expression levels of LDHA and LDHB did not increase with AdipoRon treatment in ND7/23 cells transfected with *AdipoR1/R2* siRNA, as shown by IF and WB analysis (Fig. 4C). AdipoRon treatment also resulted in increased AMP levels and decreased ATP and lactate levels but did not increase the ATP/AMP ratio in transfected ND7/23 cells (Fig. 4D). In the immunofluorescence study, the expression of Fluo-4AM did not increase, while the expression of DHE, MitoSOX, and TUNEL did not decrease in transfected ND7/23 cells, even after AdipoRon treatment (Fig. 4E). Additionally, double immunofluorescence staining with MitoSOX demonstrated that AdipoRon treatment did not reduce mitochondrial oxidative stress or increase the expression of MCT1, MCT2, or MCT4 in ND7/23 cells transfected with *AdipoR1/R2* siRNAs (Fig. 4F). This indicates that AdipoRon’s protective metabolic effects in ND/7/23 was also mediated by both adipoR1 and adipoR2 as in the hSC. Interestingly, all MCT1/2/4 expressions after AdipoRon treatment were negatively related to mitochondrial oxidative stress (Fig. 4F). Furthermore, intracellular AMP levels and ATP/AMP ratios were significantly increased and decreased, respectively, in ND7/23 cells compared to those in HSCs, regardless of the type of cellular media or the treatment with adipoRon (Fig. 4G). However, the decreased lactate and ATP/AMP concentrations in high glucose and palmitate media in hSC were increased to the level of low glucose media after AdipoRon treatment (Fig. 4G). Collectively, these results showed that the favorable effect of AdipoRon treatment worked through AdipoR1 and AdipoR2 equally. Furthermore, intracellular levels of lactate and ATP/AMP in hSCs were higher than ND7/23 cells cultured in low glucose media, which were significantly decreased in high glucose and palmitate media. On the contrary, AdipoRon treatment restored the lactate and ATP gradients between hSCs and ND7/23 cells in high glucose and palmitate media. This suggests that maintenance of gradient of lactate and ATP/AMP concentration between SCs and ND7/23 cells under diabetic conditions plays a critical role in neuroprotection in DPN by AdipoRon treatment (Fig. 4G). The use of *AdipoR1/R2* siRNAs with AdipoRon treatment clearly showed that the neuroprotective effects of AdipoRon under high-glucose and palmitate conditions are mediated equally through both AdipoR1 and AdipoR2 in hSCs and ND7/23 cells.

## Discussion

AdipoRon improved sensorimotor function and prevented peripheral nerve damage, such as fibrosis, inflammation, loss of unmyelinated fibers, axonal degeneration, and apoptotic cell loss, in the sciatic nerve and paw skin of diabetic mice. AdipoRon also maintained autophagic activity related to mitochondrial biogenesis and function while decreasing oxidative stress. It activated intracellular Ca^++^-CaMKKβ/LKB1/AMPK and decreased mTOR phosphorylation through upregulation of both AdipoR1 and AdipoR2, leading to the activation of PPARα/PGC-1α/Nrf2. These changes helped maintain the ATP/AMP ratio, a sensitive indicator of energy stress, at appropriate levels, protecting against nerve damage by increasing MCT1/2/4 expression in the sciatic nerve of diabetic mice. This suggests that AdipoRon prevents neurons and SCs in DPN from cellular energy starvation by providing lactate and ATP. Ultimately, these changes led to decreased apoptosis and increased autophagic activity, maintaining mitochondrial numbers and ensuring adequate energy interactions between neuronal axons and SCs in the sciatic nerve.

Recent studies have shown that adiponectin exerts neuroprotective effects in various CNS diseases [33, 34]. It plays a prominent role in activating adiponectin/AdipoR1 signaling to exert protective effects in the traumatic brain injury (TBI) process. It also mitigates TBI-induced mitochondrial injury and oxidative stress modulation of SIRT3-mediated protective effects on mitochondria, targeting peroxiredoxin-3 through the adiponectin/SIRT3 axis to reduce oxidative stress and promoting SIRT3 transcription through the AdipoR1/AMPK/PGC-1α signaling. However, the role of adiponectin in metabolic interactions between axons and SCs in DPN has not been studied.

The mechanisms of insulin signaling and the consequences of insulin resistance in metabolically active tissues are well-established [35]. Although, both neurons and SCs express insulin receptors, less is known about insulin resistance in peripheral nerves. PNS glucose uptake occurs via insulin-independent glucose transports (GLUTs), but insulin regulates PNS metabolism through nutrient sensors such as mTORC1 [36]; these interact with energy sensors such as AMPK and regulators of mitochondrial biogenesis such as PGC1α [37]. During nutrient excess, insulin resistance can impair insulin-mediated nutrient and energy responses [38], disrupting nerve metabolism, and leading to nerve injury. In this current study, AdipoRon treatment increased the numbers of mitochondria in the axons and SC in the sciatic nerve in diabetic mice, suggesting that it reduces ROS production in sciatic nerve mitochondria, offering a protective effect against DPN.

Neurons require significant amounts of energy to maintain membrane potential and ion gradients, making them particularly sensitive to oxidative stress [39]. Lactate was long considered a glycolytic by-product but is now recognized as a signaling molecule involved in cell survival. Under physiological conditions, neurons utilize lactate to produce and metabolize lipid; however, high levels of ROS can disrupt neuronal MCTs, prevent lactate import, reduce lipid synthesis, and aggravate neurodegeneration, leading to axonal degeneration and sensory and motor dysfunctions [9]. Lactate functions not only as a metabolic substrate but also as a signaling molecule that modulates cellular functions under pathophysiologic conditions [40]. Mice lacking one copy of MCT1 gene present normal myelin but show impaired axon regeneration after sciatic nerve crush injury [12]. MCT1-depleted SCs have reduced abilities to phagocyte myelin debris and promote axon regrowth and remyelination due to reduced energy levels and imbalanced pH and redox status [41]. Conditional knockout of MCT1 in SCs results in normal development and myelination of peripheral nerves, but with progressive thinning of myelin in sensory fibers, reduced sensory nerve conduction, and mechanical sensitivity [13]. These results suggest that the altered transport of energy-rich metabolites impairs SC function and that sensory neurons are more susceptible to metabolic imbalances, a common feature of peripheral neuropathies.

Transcriptional changes of MCTs in various tissues and disease states are associated with PPARα, AMPK, and Nrf2 [42]. MCT1 and MCT4 are closely related to AMPK in denervated muscle [43] and PPARα activation in the liver, kidney and small intestine [44]. AICAR-induced MCT1/4 up-regulation is blocked by Compound C, an AMPK inhibitor, which requires PGC-1α activation [43]. Regulation of gene transcription by PPARα is medicated via PPRE in the promoter of its target genes. Konig et al. found that PPARα activation by synthetic agonist clofibrate and by fasting increased mRNA concentration of MCT1 in the liver of rats and in rat hepatoma cells treated with WY14,643 (another selective PPARα agonist) [44]. Additionally, MCT1 expression increases with upregulation of Nrf2 [45]. AdipoRon is known to activate AMPK, PPARα and Nrf2, which increase MCT expression in various organs [19, 20, 46]. We determined that AdipoRon activates both AdipoR1 and AdipoR2, which in turn regulates MCT1/2/4 expression in the sciatic nerve, neuronal cells, and SCs under diabetic conditions. Consequently, AdipoRon ameliorated DPN in the sciatic nerve and paw skin of *db/db* mice by increasing AdipoR1 and R2 expression, subsequently activating their target molecules through the AMPK/PPARα pathway and suppressing mTOR. These changes affected downstream pathways, including PGC-1α/Nrf2, which regulated energy metabolism in nerves and SCs with the help of MCT1/2/4. The decrease in oxidative stress and an increase in mitochondrial efficiency induced by AdipoRon prevented energy deficiency-induced damage in neurons and SCs, while maintaining adequate lactate and ATP levels. Targeting energy provision under diabetic stress with AdipoRon might enhance autophagy and reduce apoptotic cell death in peripheral nerves.

Adequate intracellular lactate levels promote cellular defense mechanisms against oxidative stress by activating Nrf2 in neuroblastoma cells and the *C. elegans* [47]. Our results, along with those of previous studies, suggest that intracellular Nrf2 increases lactate levels, and vice versa, in neuronal cells and SCs. AdipoRon-induced expression of AMPK, PPARα, PGC-1α, and Nrf2 through both AdipoR1 and AdipoR2 are involved in regulating MCT1/2/4 and may support cellular functions by increasing lactate and ATP levels in the sciatic nerve in type 2 diabetic mice. These findings suggest that AdipoRon exerts anti-inflammatory, antioxidant, anti-apoptotic, and pro-autophagic effects in DPN by regulating intracellular energy, especially lactate and ATP. We hypothesize that some of the effects observed on mitochondrial dynamics might be related AdipoRon-mediated intracellular Ca^++^ balance, which affects mitochondrial dynamic-related protein activity in ROS-related neural damage [48].

**Fig. 5:**
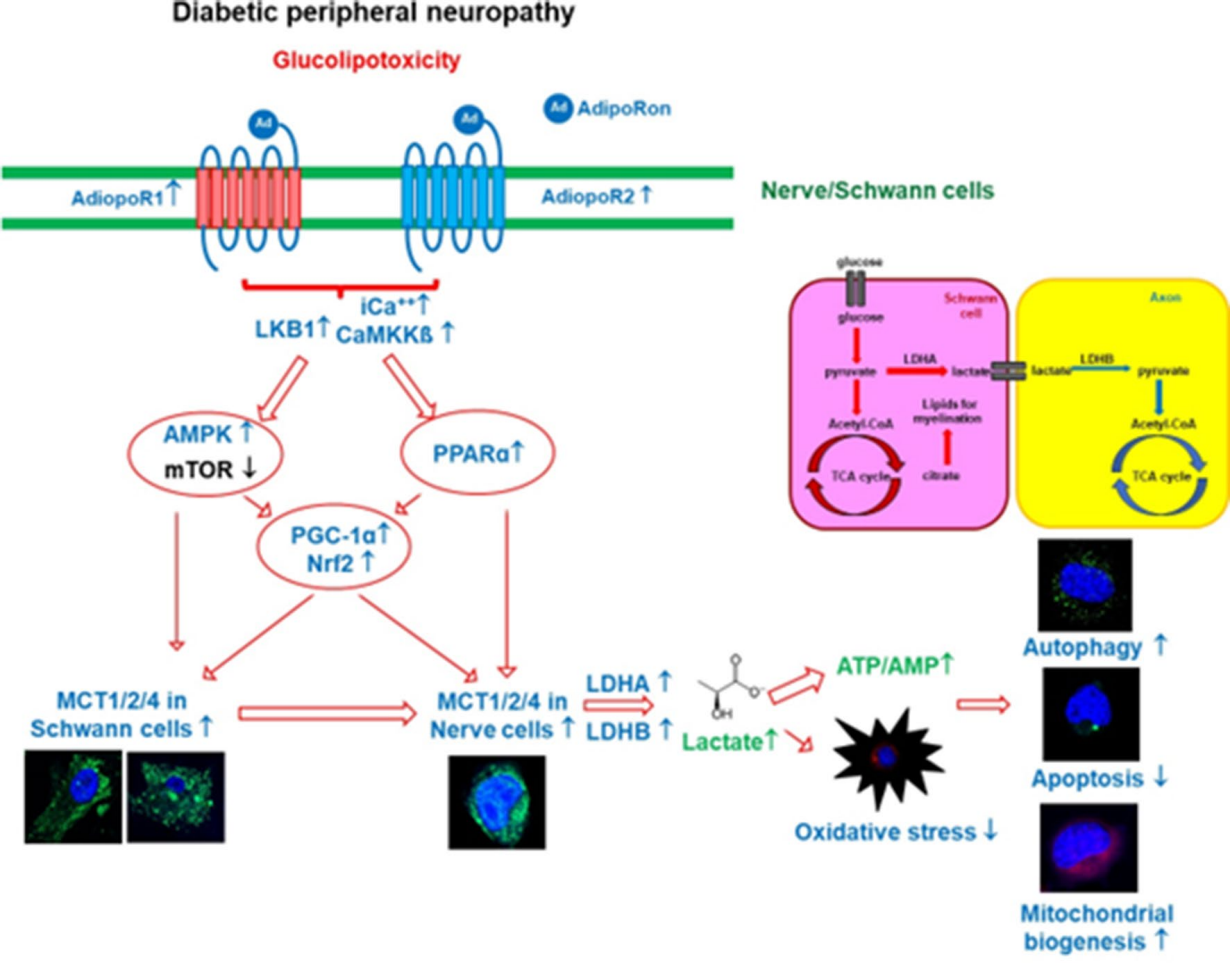
Mechanisms of AdipoRon in ameliorating diabetic peripheral neuropathy (DPN). AdipoRon enhances CaMKKβ/LKB1/AMPK/PPARα/PGC-1α/Nrf2 signaling and suppresses mTOR via increased AdipoR1 and AdipoR2 expression, promoting neuronal and Schwann cell survival, reducing apoptosis, and enhancing autophagy under diabetic conditions. Increased MCT1, MCT2, and MCT4 expression facilitates energy substrate supply (lactate and ATP) and metabolic communication between neurons and Schwann cells

In conclusion, our results indicate that AdipoRon-induced activation of AMPK/PPARα/PGC-1α/Nrf2 and suppression of mTOR through increased expression of both AdipoR1 and R2 ameliorated the development and severity of DPN. These actions contribute to neuronal cell and SC survival as well as axonal myelination by reducing apoptosis and enhancing autophagy under diabetic conditions such as high ROS levels and metabolic stress. This was achieved by providing energy substrates such as lactate and ATP through the increased expression of MCT1/2/4, facilitating metabolic communication between neurons and SCs (Fig. 5). We also identified a novel role for AdipoRon as protectant in DPN, which lead to innovative, nontoxic approaches for treating DPN. Equally important, we suggest that the maintenance of nutritional energy gradient of lactate and ATP/AMP concentration between SCs and nerve cells may be a new mechanism to provide a protective effect of nerve cells and SC against diabetes-induced neural damage. This research offers opportunities for a deeper understanding of the effects of adiponectin on neuronal function in type 2 diabetes.

## Data Availability

No datasets were generated or analysed during the current study.

## Abbreviations

AdipoR: Adiponectin Receptor
AMPK: AMP-activated Protein Kinase
ATP: Adenosine Triphosphate
CaMKK: Calcium/Calmodulin-dependent Protein Kinase Kinase
DPN: Diabetic Peripheral Neuropathy
DRG: Dorsal Root Ganglion
FFA: Free Fatty Acid
HOMA-IR: Homeostatic Model Assessment of Insulin Resistance
LDHA/LDHB: Lactate Dehydrogenase A/B
MCT: Monocarboxylate Transporter
MNCL: Motor Nerve Conduction Latency
mTOR: Mammalian Target of Rapamycin
Nrf2: Nuclear Factor Erythroid 2–Related Factor 2
PGC-1α: Peroxisome Proliferator-Activated Receptor Gamma Coactivator 1 Alpha
PNS: Peripheral Nervous System
PPAR: Peroxisome Proliferator-Activated Receptor
ROS: Reactive Oxygen Species
SC: Schwann Cell
siRNA: Small Interfering RNA
TUNEL: Terminal deoxynucleotidyl Transferase dUTP Nick End Labeling
